# Targeting the ZMIZ1-Notch1 signaling axis for the treatment of tongue squamous cell carcinoma

**DOI:** 10.1038/s41598-024-59882-y

**Published:** 2024-06-12

**Authors:** Yunqing Pang, Yunjie Sun, Yuyan Wu, Jiamin Li, Pingchuan Qin, Shanchuan Guo, Wenlian Zhou, Jian Chen, Jing Wang

**Affiliations:** 1 Lanzhou University, Lanzhou, 730000 Gansu China; 2 The First Hospital of Lanzhou University, Lanzhou, 730000 Gansu China; 3 Clinical Research Center for Oral Diseases, Lanzhou, 730000 Gansu China; 4 Clinical Education Woody L. Hunt School of Dental Medicine, Dental Medicine Texas Tech University Health Sciences Center El Paso, El Paso, Texas 79905 USA

**Keywords:** ZMIZ1, TSCC, Notch1 signaling pathway, Invasion and metastasis, Cancer genomics, Cancer therapy, Oral cancer

## Abstract

Zinc finger MIZ-type containing 1 (ZMIZ1) is a transcriptional coactivator related to the protein inhibitors of activated STATs (PIAS) family. Mounting evidence suggests that ZMIZ1 plays a crucial role in the occurrence and development of cancers. The function of ZMIZ1 in tongue squamous cell carcinoma (TSCC) and the mechanisms underpinning its role in this disease have not been fully clarified. We performed qualitative ZMIZ1 protein expression analyses using immunohistochemistry in 20 patient-derived, paraffin-embedded TSCC tissue sections. We used RNAi to knock down ZMIZ1 expression in the CAL-27 TSCC cell line and quantified the impact of ZMIZ1 knock down on proliferation, migration and apoptosis via CCK-8, scratch assay and flow cytometry, respectively. We used qRT-PCR and western blotting to investigate the role of ZMIZ1 in this cell line. Finally, we established a model of lung metastasis in nude mice to replicate the in vitro results. ZMIZ1 protein was significantly more abundant in TSCC case tissue samples. ZMIZ1 knockdown reduced the invasion and metastases of TSCC tumor cells and promoted apoptosis. ZMIZ1 knockdown was associated with the down-regulation of Notch signaling pathway related factors Jagged1 and Notch1, and invasion and metastasis related factors MKP-1, SSBP2 and MMP7 in vitro and in vivo, at the mRNA level. In vitro and in vivo data suggest that knock down of ZMIZ1 may inhibit TSCC invasion and metastasis by modulating Notch signaling. ZMIZ1 inhibition may therefore represent a new therapeutic target for TSCC.

## Introduction

Tongue squamous cell carcinoma (TSCC) is an oral squamous cell carcinoma, characterized by high risk of recurrence, metastatic, and poor prognosis^1^. TSCC predominantly manifests as moderately to well-differentiated squamous cell carcinoma originating from epithelial cells. It commonly occurs at the margin of the tongue and is associated with smoking, alcohol abuse, betel nut chewing and oral inflammation^2^. Presently, the primary treatment approach is surgical resection supplemented by a comprehensive sequential therapy combining radiotherapy, chemotherapy, and other targeted treatments^3,4^. Despite this treatment regime, TSCC exhibits early lymph node metastasis, high rates of malignancy, rapid growth, lack of early specific biomarkers, and difficult diagnosis, leading to a 5-year survival rate of only 30–50%^5,6^. Thus, urgent investigation of TSCC pathogenesis and metastasis is required at the molecular level with the aim of developing novel therapeutics.

We previously reported that ZMIZ1 and Notch1 expression were associated with the anti-proliferative agent evodiamine (EVO) treatment in CAL-27 cells by gene-chip analysis^7^. These findings suggested that Notch1 and ZMIZ1 may represent therapeutic targets for treatment of TSCC.

Notch1, a member of the Notch family, is closely associated with various types of cancers including breast^8^, leukemia^9^, colorectal ^10^, and head and neck squamous cell cancers^11^. Deng et al.^12^ used TCGA and METABRIC gene expression data to report a positive correlation between elevated Notch1 expression and the incidence of triple-negative breast cancer (TNBC). Notch1 activation compensated for BRCA1 deficiency, promoting TNBC formation and epithelial-mesenchymal transition (EMT), thereby driving TNBC metastasis and progression. Furthermore, cancers in which Notch1 is overexpressed display increased likelihood of lymph node metastasis, higher TNM staging, and significantly reduced overall patient survival rates^13,14^. Notably, in human tongue cancer, Notch1 exhibited elevated expression and promotes TSCC invasion and metastasis by modulating the expression of matrix metalloproteinases (MMPs) and epithelial-mesenchymal transition^14^. Moreover, Notch1 expression can be used as a primary biomarker to aid early diagnosis of TSCC as well as providing prognostic value^15^.

ZMIZ1 (Zinc finger MIZ-type containing 1), also known as ZIMP10 or RAI17, is a transcriptional coactivator associated with the protein inhibitors of activated STATs (PIAS) family. ZMIZ1 expression is elevated in various tumors and is linked to clinical characteristics such as age of onset, progression, malignancy, prognosis, and chemotherapy resistance^16,17^. ZMIZ1 was reported to regulate Notch1 expression, targeting the Hes1 and Myc genes to modulate precursor T-cell development^18^. Furthermore, ZMIZ1 is involved in transcriptional activation of Notch1 target genes ^17^. Through direct interaction with Notch1, ZMIZ1 was commonly co-expressed with activated Notch1 in T-cell acute lymphoblastic leukemia (T-ALL) ^18^. Knockout of ZMIZ1 inhibited the occurrence of Notch1-induced leukemia, and it was proposed that targeting ZMIZ1 could treat lymphocytic leukemia^19,20^. Together, these studies suggest that ZMIZ1 might act as a direct transcriptional co-factor for Notch1, and disrupting the Notch1-ZMIZ1 interaction may impact upon tumorigenesis, proliferation and metastasis in various cancers.

In this study, we aimed to explore the ZMIZ1-Notch1 axis as a potential therapeutic strategy in TSCC. We clarified the role of ZMIZ1 in the occurrence and development of TSCC. We knocked down ZMIZ1 in TSCC tumor cells and investigated the impact on TSCC invasion and metastasis in vitro and in vivo. Then, we detected the expression of invasion and metastasis related factors to explore the molecular mechanisms linking ZMIZ1 and Notch1, include mitogen-activated protein kinase phosphatase-1 (MKP-1), single-stranded DNA binding protein 2 (SSBP2) and matrix metalloproteinase 7 (MMP7).

## Materials and methods

### Patients and clinical specimens

Twenty specimens of human TSCC embedded in paraffin were used. The specimens were collected from the first and second hospitals of Lanzhou University (Gansu, China). The basic clinical information of TSCC patients is shown in Supplementary Table 1. The informed consent forms were duly signed by and collected from all patients. This study was approved by the Medical Ethics Committee of Lanzhou University School of Stomatology (Gansu, China). And all authors confirmed that methods used were carried out in accordance with relevant guidelines and regulations.

### Reagents

LV-ZMIZ1-RNAi was designed by Shanghai Genechem Co., LTD. (Shanghai, China). Antibodies against ZMIZ1, Jagged1, Notch1, MKP-1, SSBP2 and MMP7 were procured from Abcam (MA, USA). Antibodies against GAPDH were purchased from ImmunoWay (Texas, USA). Secondary antibody was purchased from Wuhan Boster Company (Wuhan, China). All other reagents and compounds were of analytical grade and purchased from local chemical suppliers in China.

### Cell culture and transfection

Human TSCC cell line CAL-27 was purchased from ATCC (https://www.atcc.org/products/all/CRL-2095.aspx). STR analysis was performed in this cell line to ensure the authentication of human cell lines, and was cultured in a humidified CO_2_ incubator at 37 °C. Cells were maintained in Dulbecco's modified Eagle medium (DMEM) containing 10% fetal bovine serum (FBS) and 100 U/mL penicillin and 100 U/mL streptomycin. According to the manufacturer's protocol, LV-ZMIZ1-RNAi was used for cell transfection. CAL-27 cells were seeded into a six-well plate (3–5 × 10^4^ cells per well), and cultured in 2 mL complete medium until cells were 60% to 80% confluent. Then, the medium was changed to 1.8 mL complete medium and 200 μL LV-ZMIZ1-RNAi complex (containing 2 μl polybrene), and the transfection was confirmed under fluorescence microscope after 72 h selection with puromycin.

### RNA extraction and quantitative real-time polymerase chain reaction (qRT-PCR)

According to the reagent instructions, total RNA in cells and tissues was extracted using TRIzol reagent (Invitrogen). RNA was reverse-transcribed into cDNA using Takara Reverse Transcription Kit (Takara Bio Inc.), and qRT-PCR was performed using SYBR Premix Ex Taq II (Takara Bio Inc.). The relative gene expression level was calculated according to the 2^-ΔΔCT^ method, and the relative expression level of mRNA was standardized using GAPDH. Table 1 listed all primers and related sequences used in the experiment.

**Table 1:** The primer sequences used in this study.

Gene name	Sequence (5′–3′)	Tm	Amplicon length(bp)
ZMIZ1	F: CTGGAGGTGGATCAGTACATGTGG	65	24
	R: GACATTGGGCATGATCATCTGG	64.8	22
Jagged1	F: AACGGTGCCCAGTGCTACAA	59.9	20
	R: GCCACTGTGCAGCTGTCAATC	59.8	21
Notch1	F: AATGTGGATGCCGCAGTTG	63.1	19
	R: ATCCGTGATGTCCCGGTTG	64.1	19
Hes-1	F: GGACATTCTGGAAATGACAGTGA	54.6	23
	R: AGCACACTTGGGTCTGTGCTC	60.5	21
GAPDH	F: GCACCGTCAAGGCTGAGAAC	63.3	20
	R: TGGTGAAGACGCCAGTGGA	64	19

### CCK-8 assay

TSCC cell proliferation was detected by CCK-8 analysis. TSCC cells were inoculated into 96-well plates at 2000 cells/well, and 10 µL CCK-8 solution was added to each well after 24, 48, 72, 96 and 120 h as per the manufacturer’s instructions (Dojindo China CO., Ltd, Shanghai, China). The absorbance value was measured at 450 nm after culturing at 37 °C for 2 h.

### Wound healing assay

CAL-27 cells were inoculated in a six-well plate with DMEM containing 10% FBS until confluent. The cells were scraped with a sterile 200 μL pipette head to form an artificial wound. Three random images in each field were obtained with inverted microscope at 0, 24, 48, and 72 h after injury to assess wound healing. The average migration rate of cells of each group was calculated using ImageJ software.

### Annexin V-APC apoptosis detection

Annexin V-APC Cell Apoptosis Detection Kit (Biolab Technology Co., Ltd., Beijing, China) was used to determine the proportion of apoptotic cells in each group. The measurements were made according to the manufacturer's instructions. Cells were digested with EDTA-free trypsin. The collected cells were placed in a centrifuge tube, centrifuged and washed twice in pre-cooled PBS, and suspended in 500 μL 1× binding buffer, then incubated with 5 μL of Annexin V-APC. Cells were protected from light at room temperature for 10 min. Apoptotic cells were detected by FACsCalibur (BD Bioscience, San Jose, CA, USA).

### Western blots

CAL-27 cells were cultured for 48 h following transfection, and whole protein was extracted using RIPA lysis buffer (Thermo Fisher Scientific) and quantified with the BCA kit (Beyotime Biotechnology). The sample (10 μg) was collected, separated by 10% SDS-PAGE, and transferred to PVDF membrane. Then, under gentle agitation, the membrane was blocked at 37 °C for 2 h. After that, primary antibody was added to incubate at 4 °C overnight, and secondary antibody was added to incubate at 37 °C for 2 h. Chemiluminescence was detected by ultra-sensitive chemiluminescence fluid (Millipore), and then processed and analyzed by a chemiluminescence imaging system. GAPDH was used as an internal reference protein. The antibodies and concentrations used in this experiment were as follows: ZMIZ1 (1:500; Abcam); Jagged1 (1:1000; Abcam); Notch1 (1:1000; Abcam); MKP-1 (1:1000; Abcam); SSBP2 (1:1000; Abcam); MMP7 (1:1000; Abcam); GAPDH (1:5000; ImmunoWay) and HRP-conjugated secondary antibody (1:10,000; Boster). The band density was analyzed using ImageJ software. All fold changes of band density were normalized to the control group.

### In vivo metastasis assay

For experimental purposes, all nude mice experiments were conducted in accordance with the “Guidelines for Animal Care and Use” (NIH), the ARRIVE (Animal Research: Reporting of In Vivo Experiments) guidelines, and were approved by the Medical Ethics Committee of Lanzhou University School of Stomatology. Male thymus free BALB/C nude mice (nu/nu) aged 4–6 weeks were purchased from Beijing Vital River Laboratory Animal Technology Co., Ltd. (Beijing, China). Mice were raised in a laminar flow cabinet free of specific pathogens, and had free access to food and high-pressure water with a dark/light cycle every 12 h. Nude mice were randomly divided into two groups (NC control group, LV-ZMIZ1 group). For the LV-ZMIZ1 group, 200 μL of 1 × 10^7^/mL CAL-27 cell suspension was injected through the tail vein (n = 8 animals in each group). Food, water consumption and body weight were measured every three days. At the 8th week after tail vein injection, mice were sacrificed, lung tissues were dissected and photographed, lung histopathological sections were used for hematoxylin–eosin staining (HE) staining and immunohistochemistry (IHC), and the numbers of lung tumor metastases were counted.

### Hematoxylin–eosin (HE) staining

Lung samples were extracted from control and LV-ZMIZ1 lung metastasis models for HE staining. The lung tissue was fixed in 10% formalin for 24 h, embedded in paraffin, cut to 4 μm thickness, stained with HE, dehydrated, transparent, dry, and sealed with neutral gum. Each section was observed under the microscope (Leica Microsystems).

### Immunohistochemistry (IHC)

In brief, the sections from the specimens of human TSCC and the lung samples of nude mice were dewaxed and dehydrated. Endogenous peroxidase activity was blocked with 3% methanol hydrogen peroxide solution for 20 min, and non-specific immune-reactivity was eliminated by 10% normal goat serum for 30 min. Then, the tissue sections were incubated with the primary antibody. Antibodies were anti-ZMIZ1 (1:100, Abcam), anti-Jagged1 (1:200, Abcam), anti-Notch1 (1:200, Abcam), anti-MKP-1 (1:200, Abcam), anti-SSBP2 (1:200, Abcam), anti-MMP7 (1:200, Abcam). For the negative control, the primary antibody was replaced by PBS. The specimens were washed 3 times with PBS, and incubated with the goat anti-rabbit secondary antibody (ZSGB-Bio) labeled with peroxidase polymer at room temperature until positive brown staining appeared. Samples were then incubated with diaminobenzidine, counterstained with hematoxylin, dehydrated and covered with glass slides. Sections were observed under the microscope. IHC was quantitatively analyzed using Image J software, with average optical density (AOD) serving as the intensity of staining. The “Integrated density” and “Area” tools were used. AOD = Integrated density/Area. The detailed information has been added in our article.

### Statistical analysis

All the experiments were repeated at least three times. The data are expressed as mean ± standard deviation (SD). Statistical comparisons were performed by student T test or one-way ANOVA as implemented in the GraphPad Prism software (version 6.0, GraphPad). *P* values < 0.05 was considered statistically significant.

## Results

### ZMIZ1 expression is elevated in TSCC tissue

**Figure 1:**
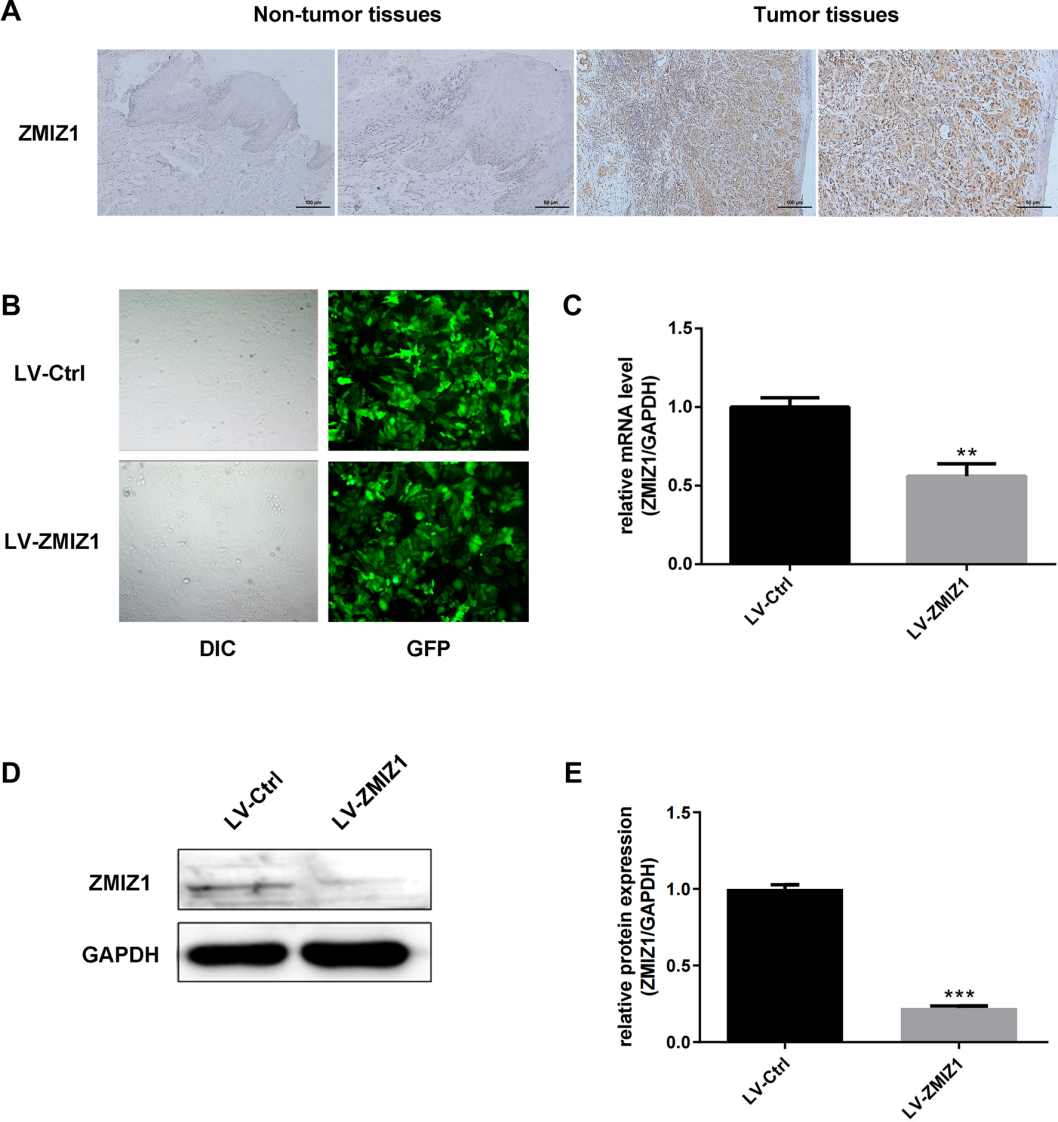
The expression of ZMIZ1 in TSCC tissues and establishment of TSCC cell lines with ZMIZ1 stable knockdown. (**A**) The expression of ZMIZ1 in TSCC tissues and adjacent non-tumor tissues were detected by IHC. (**B**) Fluorescence microscopy was used to detect the transfection efficiency of lentivirus with GFP in CAL-27 cells. (**C**) qRT-PCR was used to detect the expression of ZMIZ1 mRNA in CAL-27 cells after knockdown of ZMIZ1 mRNA. (**D**, **E**) Western blot was used to detect the expression of ZMIZ1 protein in CAL-27 cells after ZMIZ1 knockdown. The data shown represent the mean ± SD. *P < 0.05, **P < 0.01, ***P < *0.001.*

We first aimed to characterize ZMIZ1 protein expression in samples derived from TSCC patients. The protein abundance levels of ZMIZ1 were assessed through IHC in 20 paraffin-embedded TSCC tissue sections. ZMIZ1 demonstrated positive expression in both the cell membrane and cytoplasm. ZMIZ1 protein expression appeared to be abundant within the TSCC tumor tissue, but weakly expressed in adjacent non-tumor tissue (Fig. 1A and Fig. S1).

Having confirmed expression of ZMIZ1 protein in TSCC samples, we investigated the specific role of ZMIZ1 in TSCC. We established CAL-27 cell lines transfected with LV-ZMIZ1-RNAi (experimental group) and LV-Ctrl-RNAi (control group). Lentiviral vectors were used to construct LV-ZMIZ1-RNAi and LV-Ctrl-RNAi, then transfected into CAL-27 cells. An immunofluorescence assay confirmed that the efficiency of lentivirus transfection into CAL-27 cells exceeded 80% (Fig. 1B). The expression of ZMIZ1 was then detected by qRT-PCR and western blotting. ZMIZ1 mRNA and protein levels were significantly decreased (mRNA knockdown to 56%, *P* < *0.01*; protein knockdown to 22%, *P* < *0.01*) in the LV-ZMIZ1-RNAi samples compared to controls (Fig. 1C–E).

### Knockdown of ZMIZ1 inhibits proliferation and migration, and promotes apoptosis in CAL-27 cells

**Figure 2:**
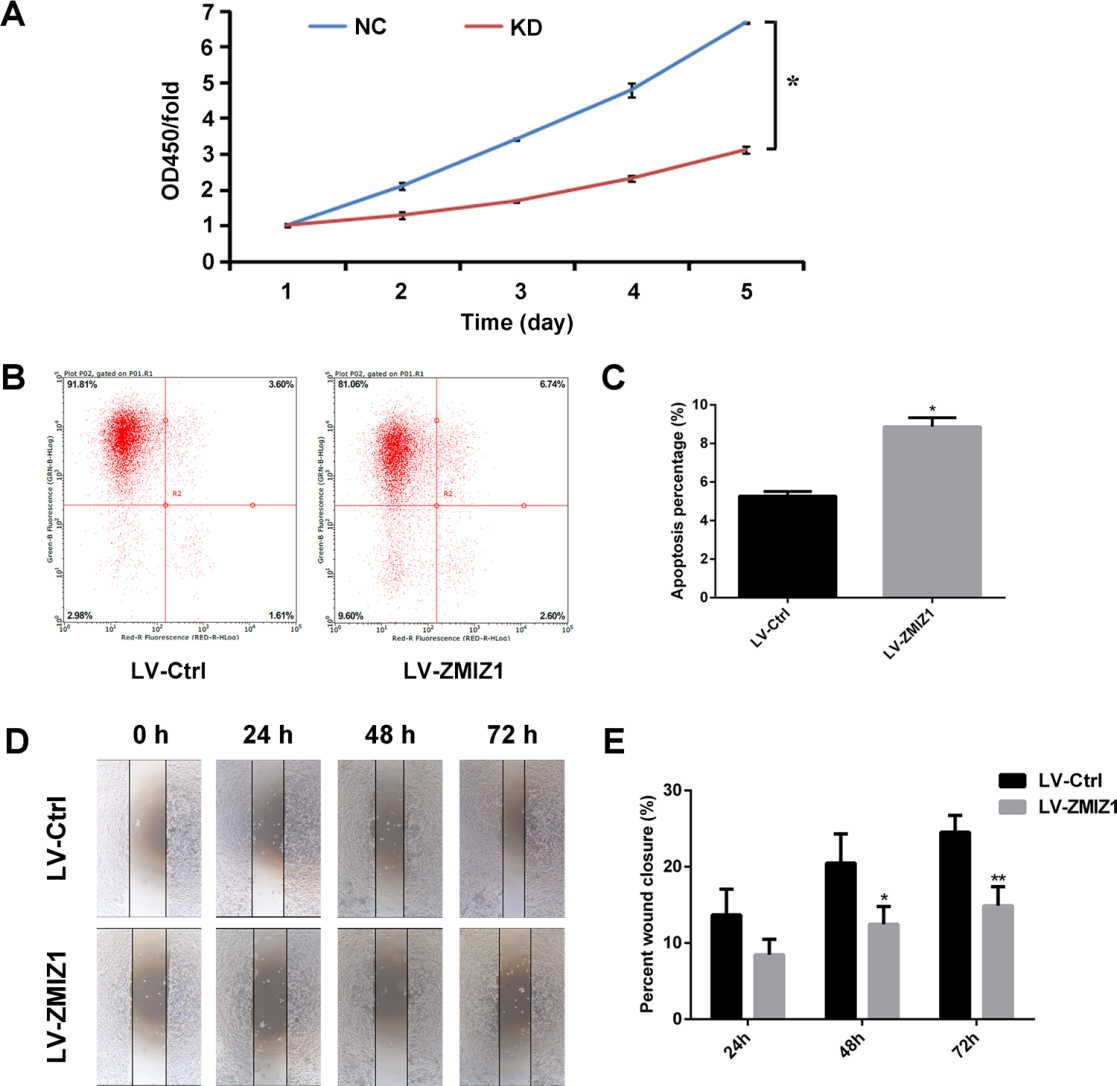
The effect of LV-ZMIZ1-RNAi on proliferation, apoptosis and migration of CAL-27 cells in vitro. (**A**) CCK-8 was used to quantify the effect of LV-ZMIZ1-RNAi on the proliferation ability of CAL-27 cells in vitro. NC = control, KD = ZMIZ1 knockdown. (**B**) Flow cytometry was used to detect the effect of LV-ZMIZ1-RNAi on the apoptosis of CAL-27 cells in vitro. (**C**) Quantitative analysis of the apoptosis of CAL-27 cells. (**D**) Wound healing assay showed the migration ability of cells in the two groups at 0, 24, 48 and 72 h, respectively. (**E**) Quantitative analysis of cell migration width in wound healing experiment. The data shown represent the mean ± SD. *P < 0.05, **P < *0.01.*

Having established a ZMIZ1-knockdown model in CAL-27 cells, we aimed to verify the role of ZMIZ1 in proliferation, apoptosis, and migration in CAL-27 cells. The CCK-8 assay was used to assess the proliferation ability of CAL-27 cells after ZMIZ1 was knocked down. The proliferation of the ZIMZ1 knockdown group was significantly inhibited compared with the control group (Fig. 2A; *P* < *0.05*).

The proportion of apoptotic cells in each group was measured by flow cytometry (Annexin V-APC single staining), and the peak value of apoptosis and the percentage of apoptotic cells were obtained. The proportion of apoptotic cells was increased in the ZMIZ1 knockdown cells, linking ZMIZ1 to apoptosis in these cells (*P* < *0.05*; Fig. 2B,C).

To investigate the role of ZMIZ1 in migration in CAL-27 cells we performed a wound healing assay. The results suggested that the scratch healing rate of ZMIZ1 knockdown cells was significantly lower than the control cells at 48 h (*P* < *0.05*), and was more even pronounced at 72 h. This indicated that the migration ability of CAL-27 cells was inhibited after ZMIZ1 was knocked down (Fig. 2D,E).

### ZMIZ1 regulates Notch1 signalling to control CAL-27 cell invasion, metastasis and apoptosis

**Figure 3:**
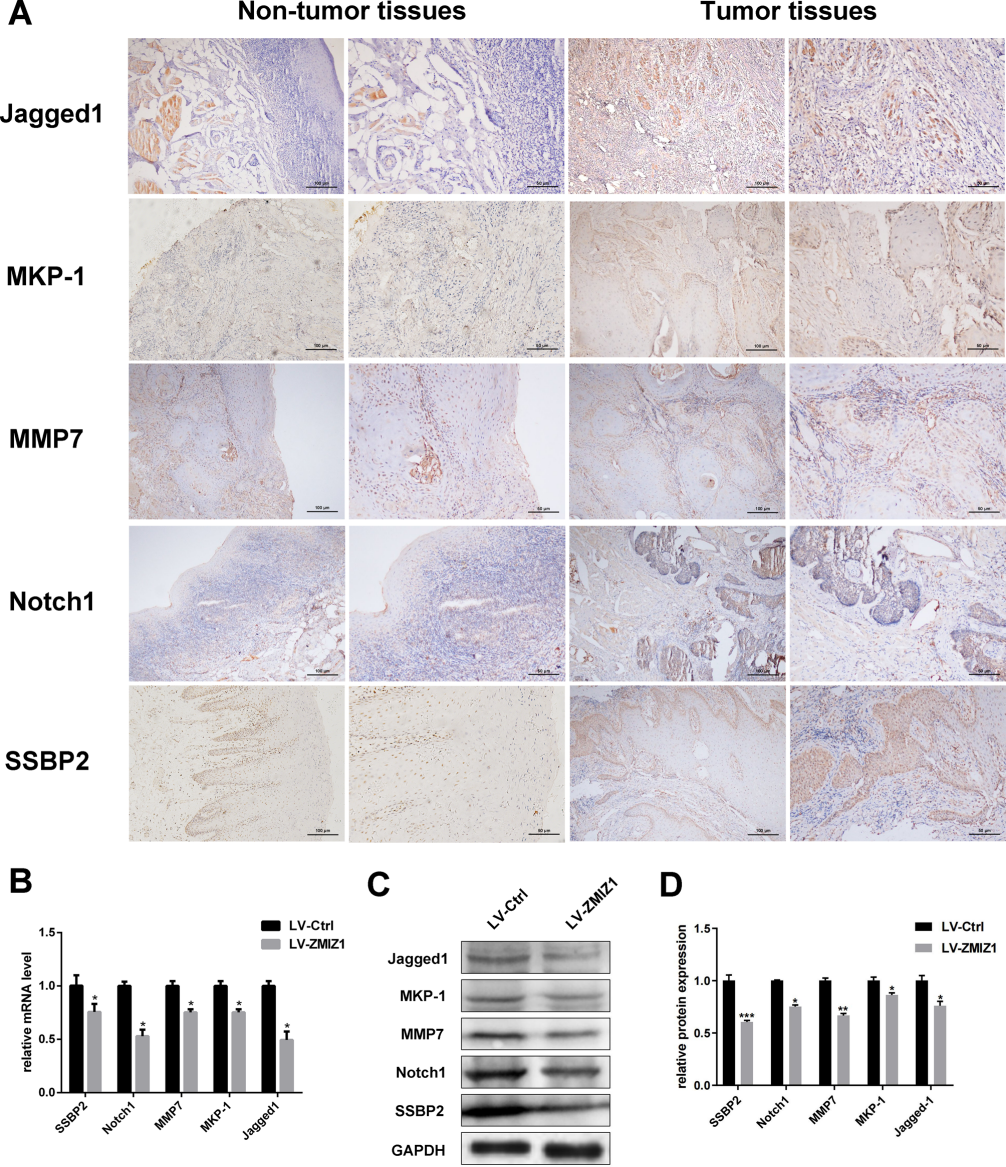
The potential mechanism of ZMIZ1 regulation of invasion and metastasis in CAL-27 cells. (**A**) IHC was used to detect the protein expression of Notch1, Jagged1, MKP-1, SSBP2, MMP7 in TSCC tissues and adjacent non-tumor tissues. (**B**) qRT-PCR was used to detect the mRNA expression of Notch1, Jagged1, MKP-1, SSBP2 and MMP7 in CAL-27 cells. (**C**, **D**) Western Blot were used to detect the protein expression of Notch1, Jagged1, MKP-1, SSBP2 and MMP7 in CAL-27 cells. The data shown represent the mean ± SD. *P < 0.05, **P < 0.01, ***P < *0.001.*

Having shown that ZMIZ1 knockdown impaired the proliferation and migration of CAL-27 cells, and increased apoptosis, we investigated the mechanisms governing this behavior. Studies have shown that ZMIZ1 is an active cofactor of Notch1, and that ZMIZ1 may promote the invasion and metastasis of tumors by regulating the Notch1 signaling pathway^19,20^. We therefore investigated the expression of Notch1 signaling pathway proteins, and various markers of invasion and metastasis by IHC analysis of TSCC patient tissue sections. Our results indicated that expression of Notch1 protein and its ligand Jagged1 were significantly higher in tumor tissue compared to normal surrounding tissue (Fig. 3A and Fig. S2). Expression of invasion and metastasis-related proteins MKP-1, MMP-7, and SSBP2 were also significantly increased in TSCC tumor tissues, consistent with a propensity of tumor cells to metastasize (Fig. 3A and Fig. S2).

Having confirmed the involvement of Notch1 and metastatic factors in the patient TSCC samples, we aimed to elucidate the specific molecular mechanisms underlying the involvement of ZMIZ1 in TSCC. We employed qRT-PCR and western blot analyses to assess the expression levels of Notch1, Jagged1, MKP-1, SSBP2, and MMP7 following ZMIZ1 knockdown in the CAL-27 cell line model. The results confirmed that mRNA and protein expression levels of Notch1, Jagged1, MKP-1, SSBP2, and MMP7 were significantly decreased in the ZMIZ1 knockdown cells (Fig. 3B–D). These results suggest that that ZMIZ1 knockdown can reduce the invasion and migration potential of TSCC tumor cells by modulation of the Notch1 signaling pathway and several invasion and metastasis markers.

### ZMIZ1 regulates invasion and metastasis through the Notch1 signaling pathway in TSCC in vivo

**Figure 4:**
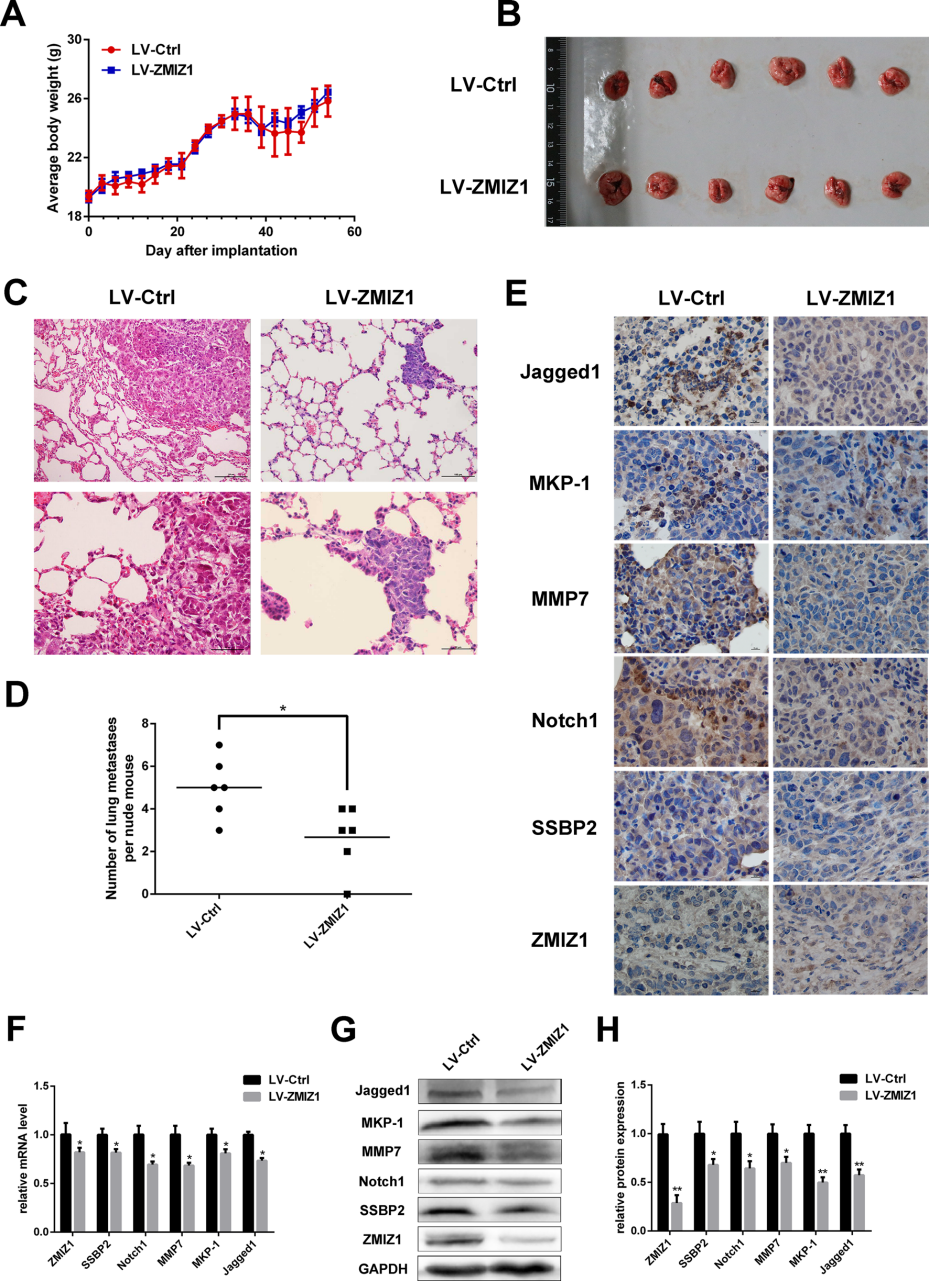
The effect of knocking down ZMIZ1 on the metastasis of TSCC in the nude mouse lung metastasis model. (**A**) After tumor cells were injected into the caudal vein, the weight change curves of nude mice were measured every 3 days. (**B**) Comparison of lung tissues of nude mice in control and the ZMIZ1 knockdown model. (**C**, **D**) HE sections and the number of lung metastases in nude mice. (E) Protein expression of Jagged1, MKP-1, MMP7, Notch1, SSBP2 and ZMIZ1 in the tissues of lung metastatic tumors in nude mice. (F-G) qRT-PCR and western blot were used to detect mRNA and protein expression of Notch1, Jagged1, MKP-1, SSBP2, MMP7 and ZMIZ1 in tumor tissues of nude mice. The data shown represent the mean ± SD. *P < 0.05, **P < *0.01.*

Following the in vitro experiments which demonstrated that the proliferation and migration of CAL-27 cells were inhibited by ZMIZ1 knockdown, we investigated the role of ZMIZ1 in TSCC metastasis in vivo. CAL-27 cells were transfected with LV-Ctrl-RNAi or LV-ZMIZ1-RNAi and were injected into the tail veins of nude mice to establish a lung metastasis model. We found no significant difference in the body weight of the mice between those inoculated with LV-Ctrl-RNAi and those with LV-ZMIZ1-RNAi (*P* > *0.05*; Fig. 4A). After 50 days, the nude mice were sacrificed and lung tissues were isolated to observe metastases under the microscope. Counting, photography and HE staining were performed. The results revealed that there were fewer lung metastases in the mice inoculated with LV-ZMIZ1-RNAi CAL-27 cells compared to the control group, and the volume of metastases was larger in the control group (*P* < *0.05*; Fig. 4B). Microscopic observation of lung metastatic tumors showed tightly arranged tumor cells with large and deep nuclei, infiltrated nuclear membranes, and distinct nucleoli in the control group. In contrast, tumors from mice with the LV-ZMIZ-RNAi CAL-27 cells displayed diffuse arrangements, appearing in streaks and clusters, accompanied by bleeding and necrosis in metastatic lesions (thickness = 4 μm, *P* < *0.05*; Fig. 4C,D). Expression levels of Jagged1, MKP-1, MMP7, Notch1, SSBP2, and ZMIZ1 in tumor tissues of LV-ZMIZ1-RNAi CAL-27 mice were decreased compared to the control group *(P* < *0.05*; Fig. 4E–H and Fig. S3). These results suggest that targeted inhibition of the ZMIZ1-Notch1 signaling pathway may reduce the expression of invasion and metastasis-related factors (MKP-1, SSBP2, and MMP7), thereby suppressing the invasion and metastasis potential of TSCC tumor cells.

## Discussion

TSCC is an oral and maxillofacial squamous cell carcinoma with high morbidity and mortality rates. Due to its location of origin, its prognosis and survival rate are poor. At present, gene-targeted therapy has become a treatment option due to its unique advantages^21^. However, it has not been utilized in the treatment of TSCC. In this study, we found that ZMIZ1 and Notch1 expression of human TSCC were significantly elevated. Knocking down ZMIZ1 in CAL-27 cells markedly reduced Notch1 expression, diminished cell proliferation and promoted apoptosis. Hence, we speculated that ZMIZ1 gene may play a regulatory role in the occurrence, development, invasion and metastasis of TSCC, and this function may be achieved by regulating the Notch1 signaling pathway. Notch1, the first Notch receptor discovered in mammals, exhibits heightened expression in various tumors, closely associated with increased cancer stem cells, tumor invasion, metastasis, and drug resistance^22^. In head and neck squamous cell carcinoma, *Notch1* is the second most commonly mutated gene after *TP53*, where inactivating mutations exert tumor-suppressive effects, and activating mutations or upregulation of expression contribute to carcinogenesis^23^. The expression of Notch1 is reportedly up-regulated in oral squamous cell carcinoma, correlating with T-stage and clinical staging^13^. Additionally, membranous Notch1 expression serves as a robust independent prognostic factor for improved outcomes in head and neck squamous cell carcinoma^24^. Our investigations show that Notch1 expression was elevated in TSCC patient tumor tissues compared to non-tumor tissues, which may be driving carcinogenesis. Consistent with our findings, Notch1 expression was upregulated in tongue cancer tissues, and inhibition of Notch1 reduced proliferation, invasion, and migration of tongue cancer cells^25^.

Targeting of Notch1 as a therapeutic strategy has undergone extensive investigation, utilizing various methods for its inhibition in cancer treatment, including Notch1 monoclonal antibodies, Notch1 siRNA, and γ-secretase inhibitors^26^. In adenoid cystic carcinoma (ACC), mutations in the *Notch1* gene which impair function correlate with higher metastatic rates and shorter survival periods. Treatment with the Notch1 monoclonal antibody bronticuzumab could delay ACC progression and contribute to partial reduction of tumor volume^27,28^. siRNA targeting Notch1 significantly reduces Notch1 expression, thereby inhibiting tumor cell proliferation and migration, inducing cell apoptosis, and exhibiting anticancer effects^29,30^. Among these approaches, research has focused on γ-secretase inhibitors (GSIs), which exert promising anticancer effects when used alone or in combination with other anticancer drugs^31^. However, anti-tumor strategies targeting Notch1 all have side effects to varying degrees. Therefore, a balanced approach is necessary where there is a continual need to explore safer approaches for targeted Notch1 inhibition in anticancer strategies.

Previous research has confirmed that ZMIZ1 may be a direct and selective co-factor of Notch1^19,32^. ZMIZ1 collaborates with Notch1 to activate c-Myc transcription and promotes M2 polarization in Kupffer cells, thereby enhancing hepatocellular carcinoma transformation^33^. However, knockdown of ZMIZ1 in Kupffer cells inhibited M2 polarization, which consequently restrained hepatocellular carcinoma progression. Similarly, in leukemia, the interaction between ZMIZ1 and Notch1 enhances C-MYC transcription and activity, facilitating leukemia initiation and progression, while ZMIZ1 inhibition attenuates leukemia progression^20^. Crucially, ZMIZ1 plays a minor role in intestinal homeostasis or bone marrow suppression. Hence, targeting ZMIZ1 can avoid the associated toxicities related to direct Notch1 targeting^19^.

Our study demonstrated that knocking down ZMIZ1 significantly suppressed the expression of Notch1 and its ligand Jagged1, confirming the correlation between ZMIZ1 and Notch1. Simultaneously, the suppression of ZMIZ1 expression inhibited the proliferation and migration of CAL-27 cells while promoting apoptosis. These findings indicated that ZMIZ1 may be a potential therapeutic target for the treatment of TSCC.

Our investigation further explored the specific mechanisms underpinning the ZMIZ1 influence on the invasion and metastasis of TSCC. Studies have shown that the expression of MMP7 was increased in tumor patients and was related to the depth of tumor invasion, size and number of lymph node metastases. MMP7 plays an important role in the malignant biological behavior of tumors^34^. Therefore, MMP7 can be used as a related effector of invasion and metastasis. Similarly, MKP-1 is overexpressed in several cancer types, associated with cancer progression, and may be a potential prognostic marker^35^. In T-ALL cell lines, cells with MKP-1 deficiency exhibited compromised tumorigenicity, which was regulated by Notch3^36^. SSBP2 is widely studied as a tumor suppressor, and downregulation of SSBP2 expression inhibited tumor cell growth and increased susceptibility of malignant tumors^37,38^. However, some studies have indicated that overexpression of SSBP2 can exert oncogenic effects which correlated with adverse clinical outcomes in hepatocellular carcinoma and glioblastoma^39,40^. Our research observed increased expression of MMP-7, MKP-1, and SSBP2 in TSCC tissue, suggesting that the invasion and metastasis of TSCC may be related to the expression of these genes. Furthermore, our results found that knockdown of ZMIZ1 correlated with decreased expression of MMP-7, MKP-1, and SSBP2, indicating that ZMIZ1 may influence the invasion and metastasis of TSCC by regulating the expression of MMP-7, MKP-1, and SSBP2.

In summary, ZMIZ1 knockdown significantly inhibits the proliferation and migration of CAL-27 cells. This mechanism potentially involves ZMIZ1 as an oncogene that activates the Notch1 signaling pathway, thereby promoting the invasion and migration of TSCC. Thus, ZMIZ1 may be a potential treatment target for TSCC.

## Conclusion

In conclusion, our investigation about the role of TSCC sheds light on its significance as a potential therapeutic target. Through a series of experiments involving ZMIZ1 knockdown, we observed a substantial inhibition in the proliferation and migration of CAL-27 cells. This suggests a pivotal role for ZMIZ1 as an oncogenic driver, possibly operating by activating the Notch1 signaling pathway, thereby facilitating TSCC invasion and migration. Additionally, our findings highlighted the association of ZMIZ1 with key factors involved in tumor progression, such as MMP7, MKP-1, and SSBP2, underscoring its multifaceted impact on TSCC biology. The identification of ZMIZ1 as a probable regulator of these invasive and metastatic factors emphasizes its potential as a promising therapeutic target for TSCC. The molecular mechanism of ZMIZ1 influencing the progression of TSCC needs to be further studied to provide new ideas for the targeted therapy of TSCC.

### Supplementary Information

Supplementary Information.

## Data Availability

The full data used to support the findings of this study are available from the corresponding author upon request.
