# Author Correction: Targeting the ZMIZ1-Notch1 signaling axis for the treatment of tongue squamous cell carcinoma

**DOI:** 10.1038/s41598-024-67609-2

**Published:** 2024-07-18

**Authors:** Yunqing Pang, Yunjie Sun, Yuyan Wu, Jiamin Li, Pingchuan Qin, Shanchuan Guo, Wenlian Zhou, Jian Chen, Jing Wang

**Affiliations:** 1 Lanzhou University, Lanzhou, 730000 Gansu China; 2 The First Hospital of Lanzhou University, Lanzhou, 730000 Gansu China; 3 Clinical Research Center for Oral Diseases, Lanzhou, 730000 Gansu China; 4 Clinical Education Woody L. Hunt School of Dental Medicine, Dental Medicine Texas, Tech University Health Sciences Center El Paso, El Paso, TX 79905 USA

Correction to: *Scientific Reports* 10.1038/s41598-024-59882-y, published online 12 June 2024

In the original version of this Article Yunqing Pang and Jing Wang were incorrectly affiliated with ‘Department of Pediatric Surgery, The First Hospital of Lanzhou University, Lanzhou 730000, Gansu, China’. The correct affiliations for Yunqing Pang and Jing Wang are listed below.

Lanzhou University, Lanzhou 730000, Gansu, China.

Clinical Research Center for Oral Diseases, Lanzhou 730000, Gansu, China.

In addition, affiliation 2 was incorrectly given as ‘Department of Pediatric Surgery, The First Hospital of Lanzhou University, Lanzhou 730000, Gansu, China’. The correct affiliation is listed below.

The First Hospital of Lanzhou University, Lanzhou 730000, Gansu, China.

The original Article has been corrected.

