# Supplementary material for: Targeting the ZMIZ1-Notch1 signaling axis for the treatment of tongue squamous cell carcinoma
Source: Sci Rep. 2024 Jun 12;14:13577. doi: 10.1038/s41598-024-59882-y (PMC11169241; doi:10.1038/s41598-024-59882-y)
Supplement: Supplementary file 1 — Supplementary Information. [file 41598_2024_59882_MOESM1_ESM.docx]

**Targeting the ZMIZ1-Notch1 signaling axis for the treatment of tongue squamous cell carcinoma**

Yunqing Pang^1,2,3†^, Yunjie Sun^1,3,†^, Yuyan Wu^1,3,†^, Jiamin Li^1,3^, Pingchuan Qin^1,3^, Shanchuan Guo^1,3^, Wenlian Zhou^4^, Jian Chen^2,*^, Jing Wang^1,2,3,*^

^1^ Lanzhou University, Lanzhou, Gansu, 730000, China

^2^ Department of Pediatric surgery, the First hospital of Lanzhou University, Lanzhou, Gansu, 730000, China

^3^ Clinical Research Center for Oral Diseases, Lanzhou, Gansu, 730000, China

4 Department of Dental Medicine, School of Dental Medicine, University of Nevada, Las Vegas, NV 89106, USA

^†^These authors contributed equally

Address correspondence to: J.W. (E-mail: [wangjing@lzu.edu.cn](mailto:wangjing@lzu.edu.cn))

J.C. (E-mail: chenjianlanyi@163.com)

Figure S1 Quantitative analysis of the expression of ZMIZ1 in TSCC tissues and adjacent non-tumor tissues were detected by IHC. The data shown represent the mean ± SD. **P<0.05, **P<0.01.*

Figure S2 Quantitative analysis of the expression of Jagged1, MKP-1, MMP7, Notch1, SSBP2 in TSCC tissues and adjacent non-tumor tissues were detected by IHC. The data shown represent the mean ± SD. **P<0.05, **P<0.01, ***P<0.001.*

Figure S3 Quantitative analysis of the expression of Jagged1, MKP-1, MMP7, Notch1, SSBP2, ZMIZ1 in the tissues of lung metastatic tumors in nude mice were detected by IHC. The data shown represent the mean ± SD. **P<0.05, **P<0.01, ***P<0.001.*

**Supplementary Table1 The clinical information of TSCC patients**

| No. | Sex | Age  (y) | Pathological number | Location | Pathological type | Antibody | The proportion of positive cells（%） | Degree of cell staining | Total IHC score |
| --- | --- | --- | --- | --- | --- | --- | --- | --- | --- |
| 1 | male | 48 | 65218499 | tongue | high differentiation | SSBP2 | 15 | light yellow | 1 |
|  |  |  |  |  |  | ZMIZ1 | 25 | deep yellow | 2 |
|  |  |  |  |  |  | Jagged1 | 20 | light yellow | 1 |
|  |  |  |  |  |  | MMP7 | 30 | deep yellow | 2 |
|  |  |  |  |  |  | MKP1 | 40 | light yellow | 2 |
|  |  |  |  |  |  | Notch1 | 45 | deep yellow | 4 |
| 2 | female | 65 | 65213807 | tongue | high differentiation | SSBP2 | 30 | light yellow | 1 |
|  |  |  |  |  |  | ZMIZ1 | 25 | light yellow | 1 |
|  |  |  |  |  |  | Jagged1 | 20 | deep yellow | 2 |
|  |  |  |  |  |  | MMP7 | 25 | light yellow | 1 |
|  |  |  |  |  |  | MKP1 | 35 | deep yellow | 4 |
|  |  |  |  |  |  | Notch1 | - | - | - |
| 3 | male | 77 | 65218499 | tongue | high differentiation | SSBP2 | 25 | light yellow | 1 |
|  |  |  |  |  |  | ZMIZ1 | 35 | deep yellow | 4 |
|  |  |  |  |  |  | Jagged1 | 30 | light yellow | 1 |
|  |  |  |  |  |  | MMP7 | 20 | tan | 3 |
|  |  |  |  |  |  | MKP1 | 55 | light yellow | 3 |
|  |  |  |  |  |  | Notch1 | 50 | light yellow | 3 |
| 4 | male | 66 | 66204137 | tongue | high differentiation | SSBP2 | 30 | light yellow | 1 |
|  |  |  |  |  |  | ZMIZ1 | 25 | tan | 4 |
|  |  |  |  |  |  | Jagged1 | 20 | deep yellow | 1 |
|  |  |  |  |  |  | MMP7 | 15 | light yellow | 3 |
|  |  |  |  |  |  | MKP1 | 55 | tan | 3 |
|  |  |  |  |  |  | Notch1 | 50 | deep yellow | 3 |
| 5 | male | 61 | 66250724 | tongue | high differentiation | SSBP2 | 25 | tan | 3 |
|  |  |  |  |  |  | ZMIZ1 | 25 | light yellow | 1 |
|  |  |  |  |  |  | Jagged1 | 30 | light yellow | 1 |
|  |  |  |  |  |  | MMP7 | 15 | light yellow | 1 |
|  |  |  |  |  |  | MKP1 | 40 | light yellow | 2 |
|  |  |  |  |  |  | Notch1 | 35 | light yellow | 2 |
| 6 | female | 71 | 65223495 | tongue | high middle differentiation | SSBP2 | 35 | light yellow | 2 |
|  |  |  |  |  |  | ZMIZ1 | 40 | light yellow | 2 |
|  |  |  |  |  |  | Jagged1 | 25 | light yellow | 1 |
|  |  |  |  |  |  | MMP7 | 45 | tan | 6 |
|  |  |  |  |  |  | MKP1 | 50 | light yellow | 2 |
|  |  |  |  |  |  | Notch1 | 55 | light yellow | 3 |
| 7 | female | 38 | 66248763 | tongue-left side | high middle differentiation | SSBP2 | 30 | light yellow | 1 |
|  |  |  |  |  |  | ZMIZ1 | 25 | light yellow | 1 |
|  |  |  |  |  |  | Jagged1 | 20 | light yellow | 1 |
|  |  |  |  |  |  | MMP7 | 15 | light yellow | 1 |
|  |  |  |  |  |  | MKP1 | 10 | tan | 3 |
|  |  |  |  |  |  | Notch1 | 15 | light yellow | 1 |
| 8 | male | 47 | 66235202 | tongue | high middle differentiation | SSBP2 | 25 | light yellow | 1 |
|  |  |  |  |  |  | ZMIZ1 | 35 | light yellow | 2 |
|  |  |  |  |  |  | Jagged1 | 30 | light yellow | 2 |
|  |  |  |  |  |  | MMP7 | 20 | deep yellow | 2 |
|  |  |  |  |  |  | MKP1 | 55 | light yellow | 3 |
|  |  |  |  |  |  | Notch1 | 50 | light yellow | 3 |
| 9 | male | 39 | 66238368 | tongue-underlying base | high middle differentiation | SSBP2 | 40 | tan | 6 |
|  |  |  |  |  |  | ZMIZ1 | 55 | light yellow | 3 |
|  |  |  |  |  |  | Jagged1 | 35 | light yellow | 2 |
|  |  |  |  |  |  | MMP7 | 15 | light yellow | 1 |
|  |  |  |  |  |  | MKP1 | 20 | light yellow | 1 |
|  |  |  |  |  |  | Notch1 | 25 | light yellow | 1 |
| 10 | male | 60 | 66242224 | tongue | middle low differentiation | SSBP2 | 15 | light yellow | 1 |
|  |  |  |  |  |  | ZMIZ1 | 5 | light yellow | 0 |
|  |  |  |  |  |  | Jagged1 | 10 | light yellow | 0 |
|  |  |  |  |  |  | MMP7 | 15 | light yellow | 1 |
|  |  |  |  |  |  | MKP1 | 15 | light yellow | 1 |
|  |  |  |  |  |  | Notch1 | 5 | light yellow | 0 |
| 11 | female | 42 | 65229864 | tongue-dorsum | middle differentiation | SSBP2 | 25 | light yellow | 1 |
|  |  |  |  |  |  | ZMIZ1 | 30 | deep yellow | 4 |
|  |  |  |  |  |  | Jagged1 | 20 | light yellow | 1 |
|  |  |  |  |  |  | MMP7 | 25 | deep yellow | 2 |
|  |  |  |  |  |  | MKP1 | 45 | light yellow | 2 |
|  |  |  |  |  |  | Notch1 | 55 | light yellow | 3 |
| 12 | male | 59 | 66235849 | tongue-right 1underlying base | middle differentiation | SSBP2 | 25 | light yellow | 1 |
|  |  |  |  |  |  | ZMIZ1 | 20 | light yellow | 1 |
|  |  |  |  |  |  | Jagged1 | 30 | light yellow | 1 |
|  |  |  |  |  |  | MMP7 | 20 | light yellow | 1 |
|  |  |  |  |  |  | MKP1 | 30 | light yellow | 1 |
|  |  |  |  |  |  | Notch1 | 25 | light yellow | 1 |
| 13 | male | 46 | 66233957 | tongue-right ventrum | middle differentiation | SSBP2 | 15 | light yellow | 1 |
|  |  |  |  |  |  | ZMIZ1 | 15 | light yellow | 1 |
|  |  |  |  |  |  | Jagged1 | 10 | light yellow | 1 |
|  |  |  |  |  |  | MMP7 | 10 | tan | 0 |
|  |  |  |  |  |  | MKP1 | 5 | light yellow | 0 |
|  |  |  |  |  |  | Notch1 | 10 | light yellow | 0 |
| 14 | female | 83 | 65205110 | tongue | middle differentiation | SSBP2 | 15 | light yellow | 1 |
|  |  |  |  |  |  | ZMIZ1 | 25 | deep yellow | 2 |
|  |  |  |  |  |  | Jagged1 | 35 | deep yellow | 4 |
|  |  |  |  |  |  | MMP7 | 20 | light yellow | 1 |
|  |  |  |  |  |  | MKP1 | 35 | light yellow | 2 |
|  |  |  |  |  |  | Notch1 | 45 | deep yellow | 4 |
| 15 | male | 65 | 66227103 | lingual surface of epiglottis | middle differentiation | SSBP2 | 20 | light yellow | 1 |
|  |  |  |  |  |  | ZMIZ1 | 35 | deep yellow | 4 |
|  |  |  |  |  |  | Jagged1 | 15 | light yellow | 1 |
|  |  |  |  |  |  | MMP7 | 35 | tan | 6 |
|  |  |  |  |  |  | MKP1 | 15 | light yellow | 1 |
|  |  |  |  |  |  | Notch1 | 25 | light yellow | 1 |
| 16 | male | 51 | 66219270 | tongue | middle differentiation | SSBP2 | 25 | light yellow | 1 |
|  |  |  |  |  |  | ZMIZ1 | 35 | light yellow | 2 |
|  |  |  |  |  |  | Jagged1 | 30 | deep yellow | 4 |
|  |  |  |  |  |  | MMP7 | 20 | deep yellow | 2 |
|  |  |  |  |  |  | MKP1 | 55 | light yellow | 3 |
|  |  |  |  |  |  | Notch1 | 50 | light yellow | 3 |
| 17 | female | 72 | 66208534 | tongue | middle differentiation | SSBP2 | 25 | light yellow | 1 |
|  |  |  |  |  |  | ZMIZ1 | 35 | light yellow | 2 |
|  |  |  |  |  |  | Jagged1 | 30 | light yellow | 2 |
|  |  |  |  |  |  | MMP7 | 20 | deep yellow | 2 |
|  |  |  |  |  |  | MKP1 | 55 | light yellow | 3 |
|  |  |  |  |  |  | Notch1 | 50 | light yellow | 3 |
| 18 | male | 56 | 66206420 | ventrum of tongue | middle differentiation | SSBP2 | 25 | light yellow | 1 |
|  |  |  |  |  |  | ZMIZ1 | 35 | light yellow | 2 |
|  |  |  |  |  |  | Jagged1 | 30 | light yellow | 2 |
|  |  |  |  |  |  | MMP7 | 20 | deep yellow | 2 |
|  |  |  |  |  |  | MKP1 | 55 | light yellow | 3 |
|  |  |  |  |  |  | Notch1 | 50 | tan | 9 |
| 19 | female | 48 | 66191601 | tongue | middle differentiation | SSBP2 | 25 | light yellow | 1 |
|  |  |  |  |  |  | ZMIZ1 | 35 | light yellow | 2 |
|  |  |  |  |  |  | Jagged1 | 30 | light yellow | 2 |
|  |  |  |  |  |  | MMP7 | 20 | deep yellow | 2 |
|  |  |  |  |  |  | MKP1 | 55 | light yellow | 3 |
|  |  |  |  |  |  | Notch1 | 50 | light yellow | 3 |
| 20 | male | 60 | 65218499 | tongue | carcinoma in situ | SSBP2 | 25 | tan | 3 |
|  |  |  |  |  |  | ZMIZ1 | 25 | light yellow | 1 |
|  |  |  |  |  |  | Jagged1 | 30 | light yellow | 1 |
|  |  |  |  |  |  | MMP7 | 15 | light yellow | 1 |
|  |  |  |  |  |  | MKP1 | 40 | light yellow | 2 |
|  |  |  |  |  |  | Notch1 | 35 | light yellow | 2 |

***** The assignment of score system

1. The proportion of positive cells（%）score: 0 = “< 10”, 1 = “10-30”, 2 = “30-50”, 3 = “> 50”.

2. Degree of cell staining score: 0 = No coloration, 1 = light yellow, 2 = deep yellow, 3 = tan.

3. Total IHC score = The proportion of positive cells（%）score * Degree of cell staining score.

**Original images for gels and blots**

**Figure 1 D**

**
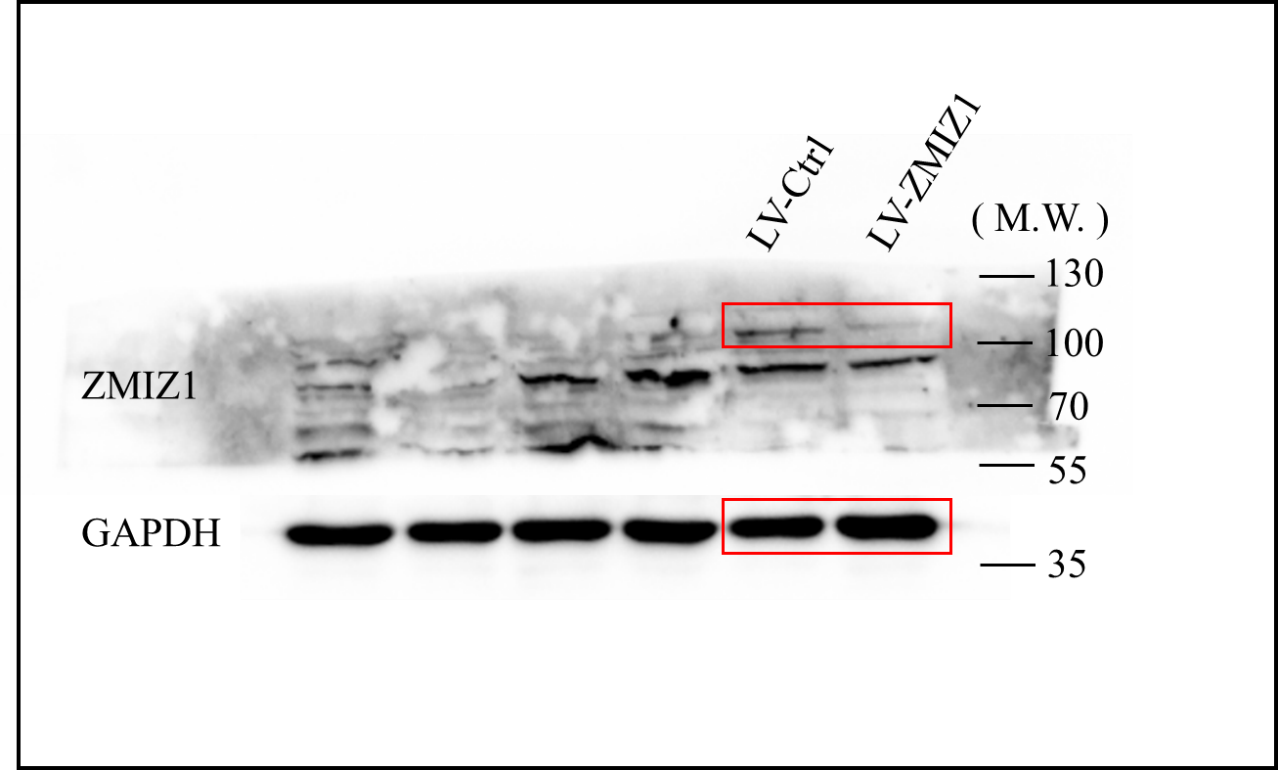
**

**
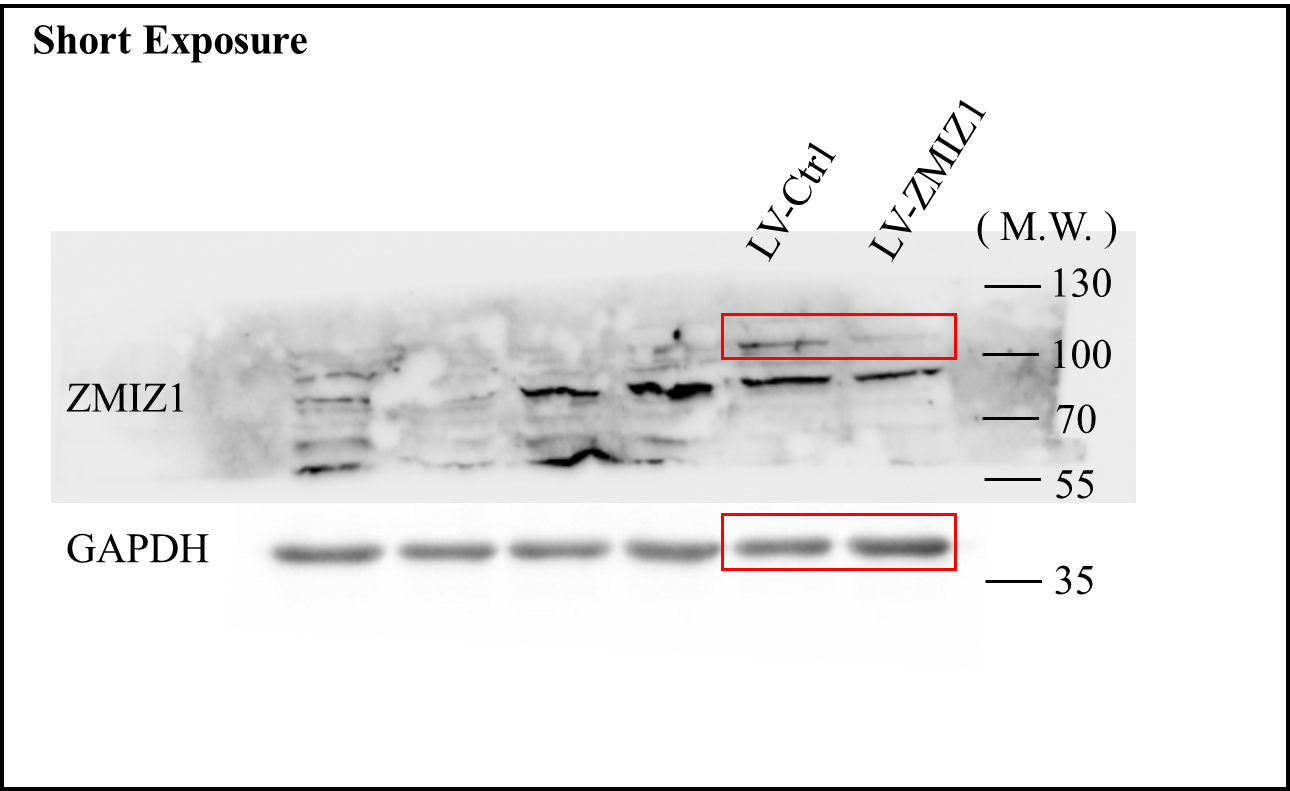
**

**Figure 3 C**

**
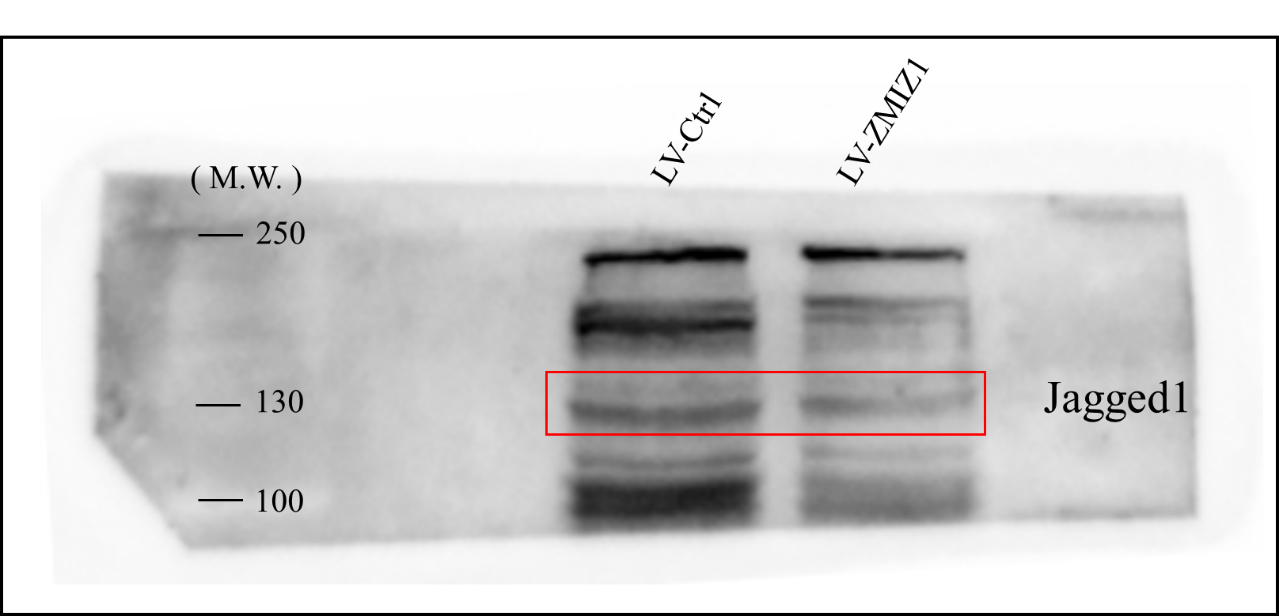
**

**
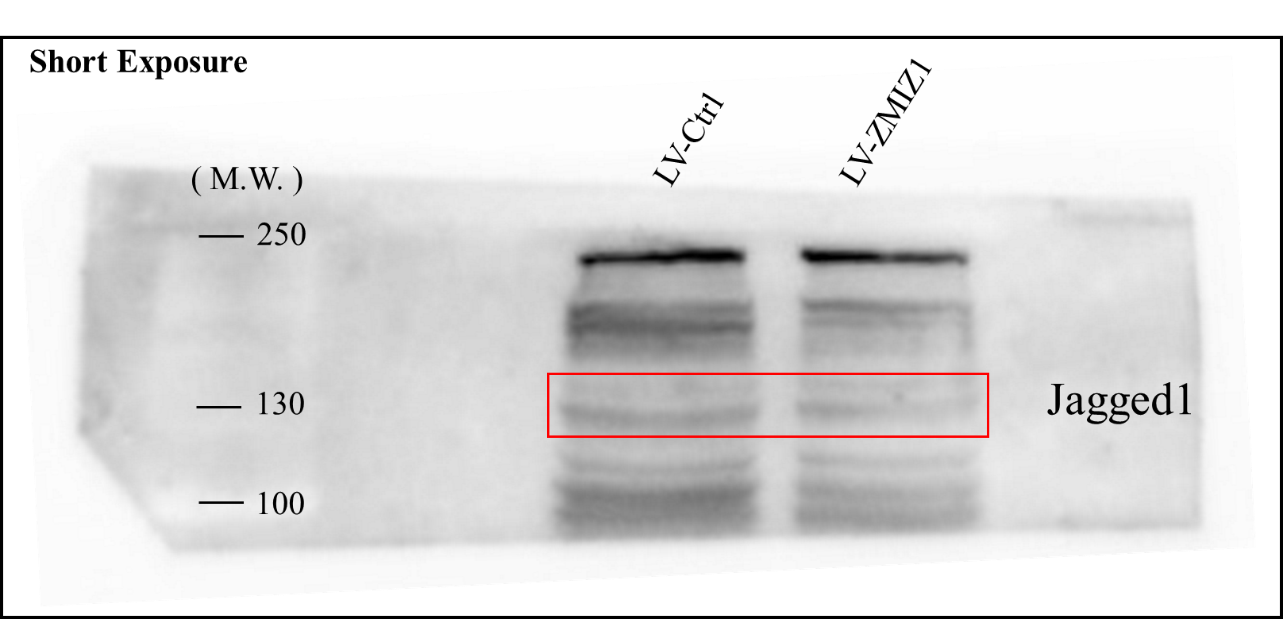
**

**
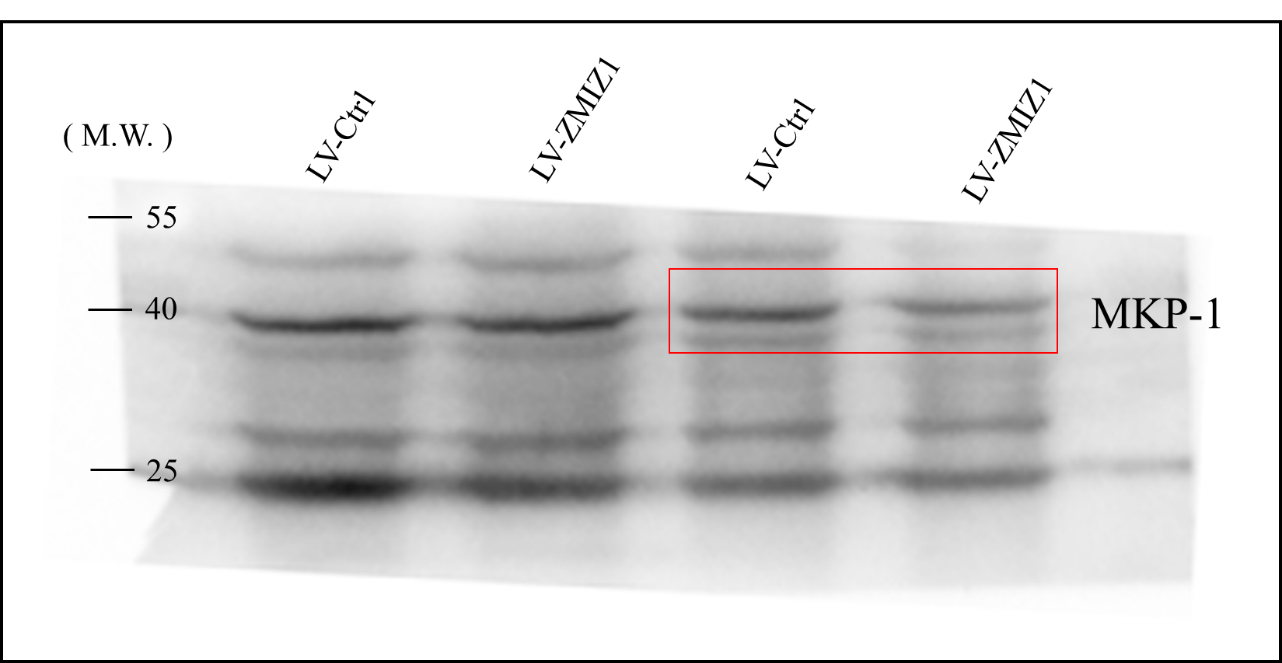
**

**Figure 3 C**

**
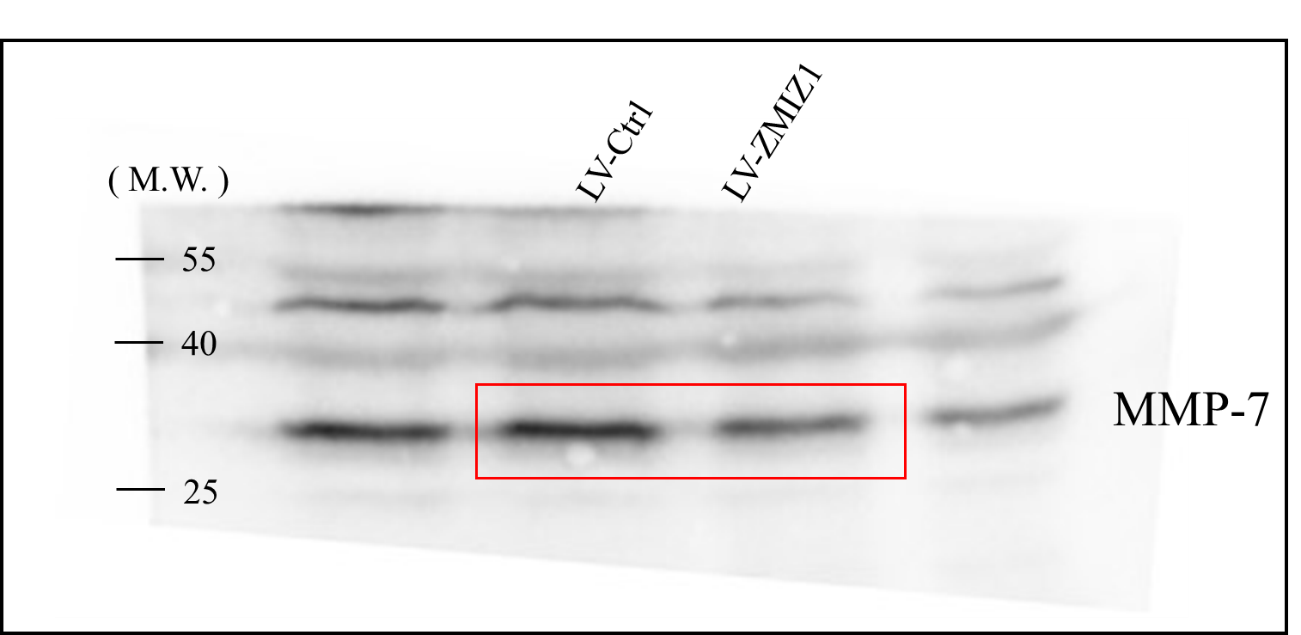

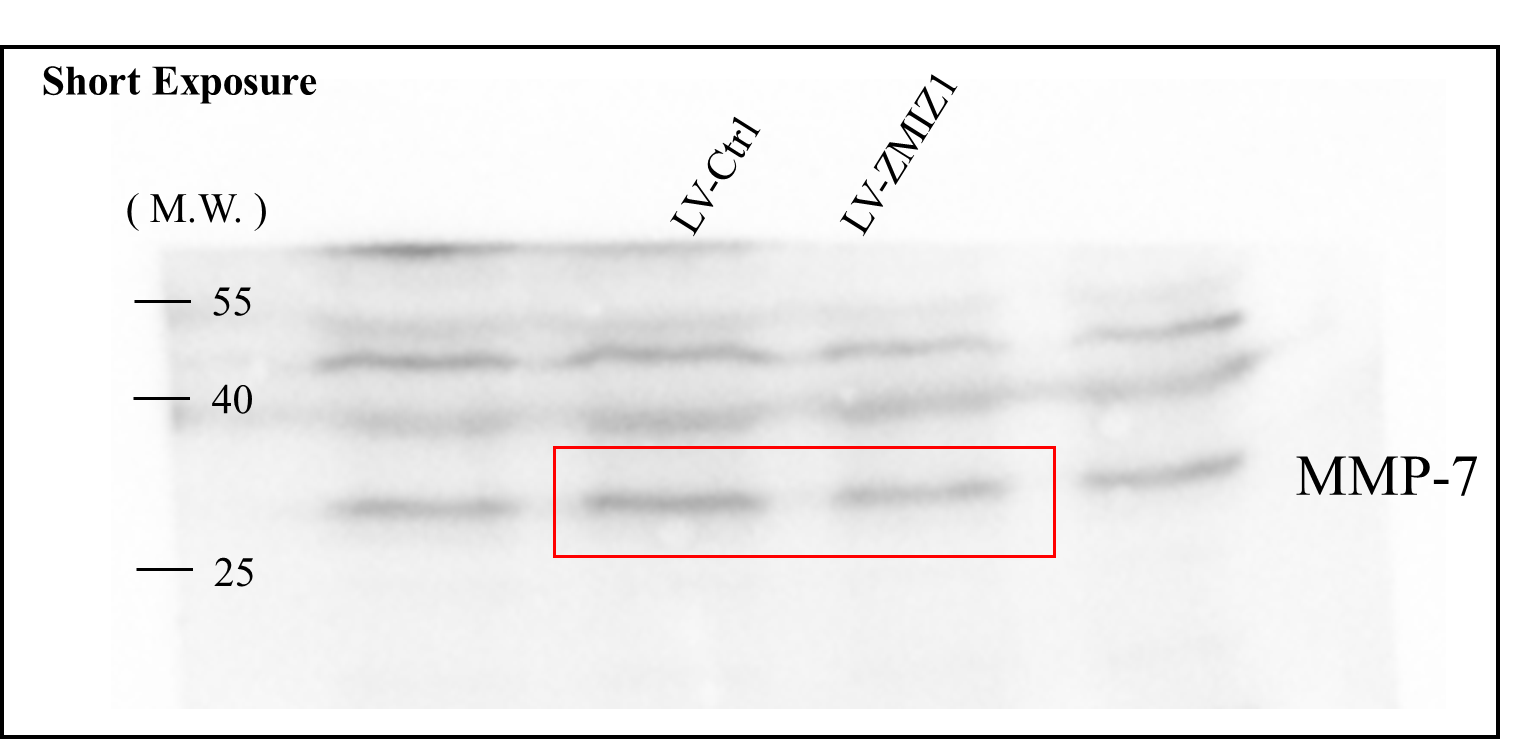
**

**Figure 3 C**

**
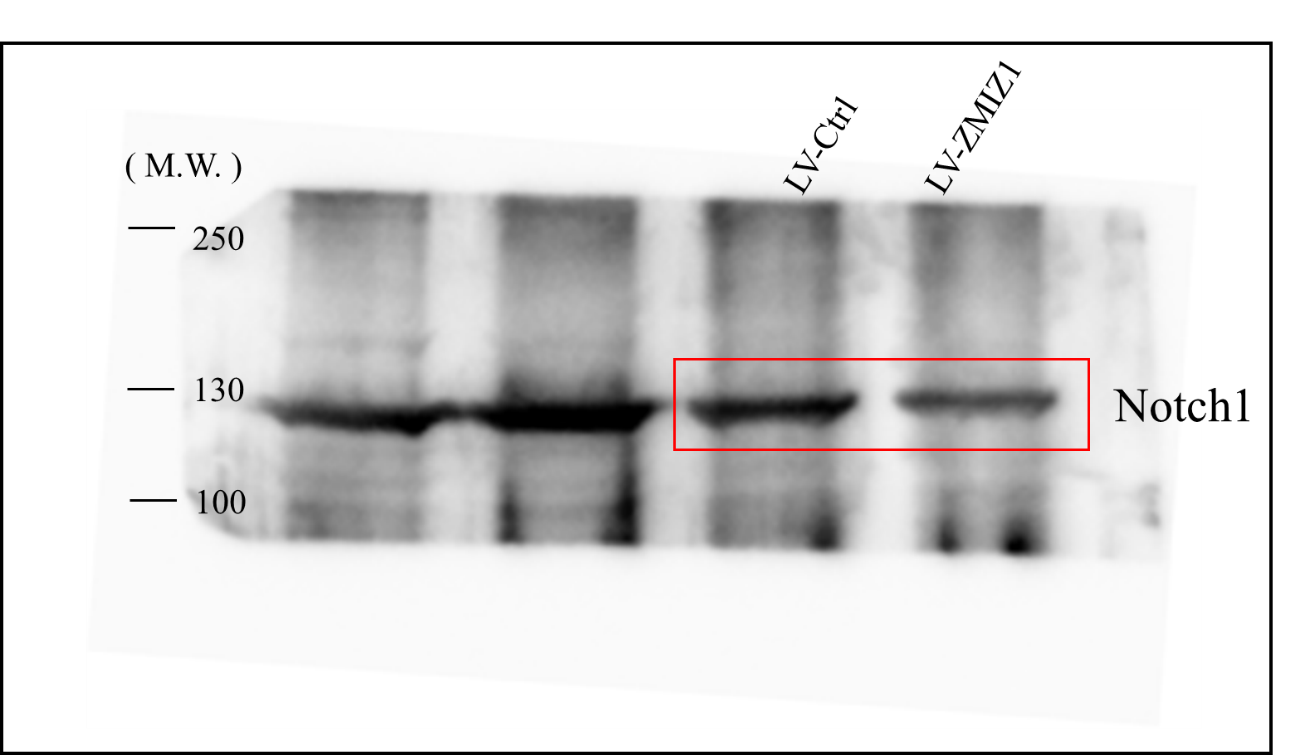

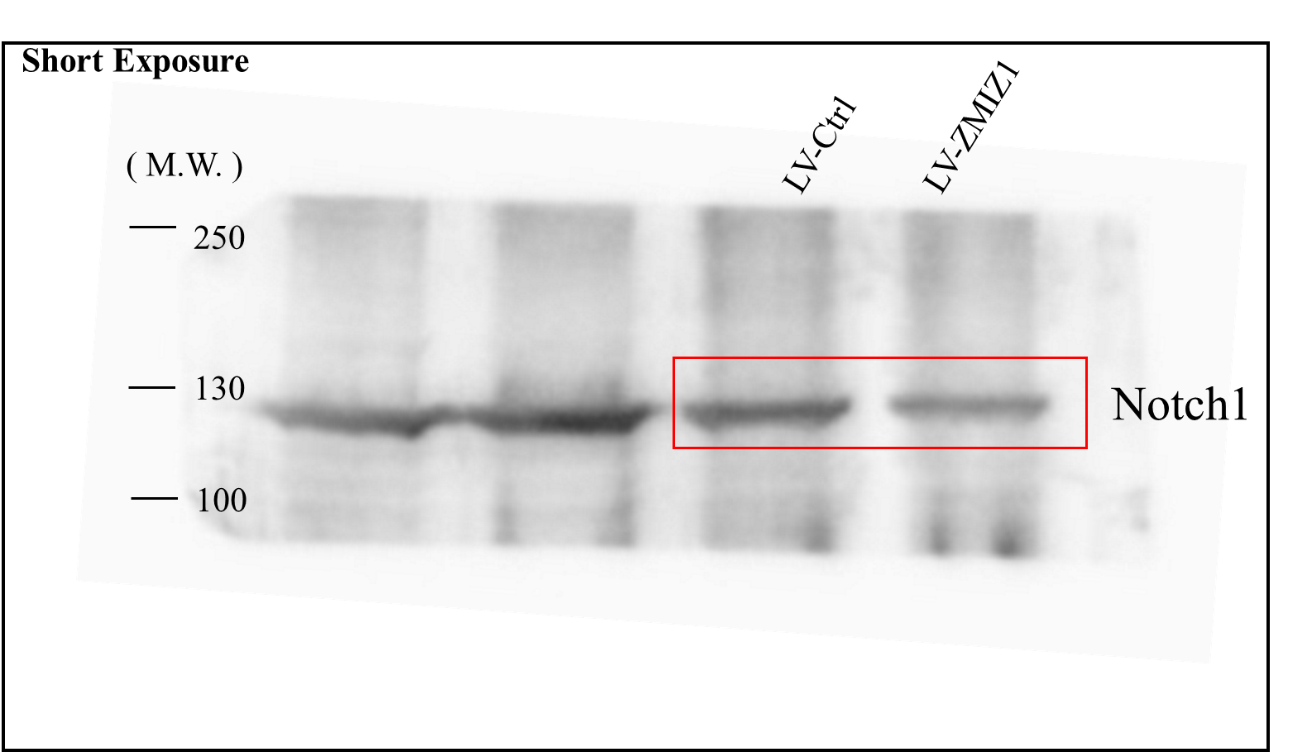
**

**Figure 3 C**

**
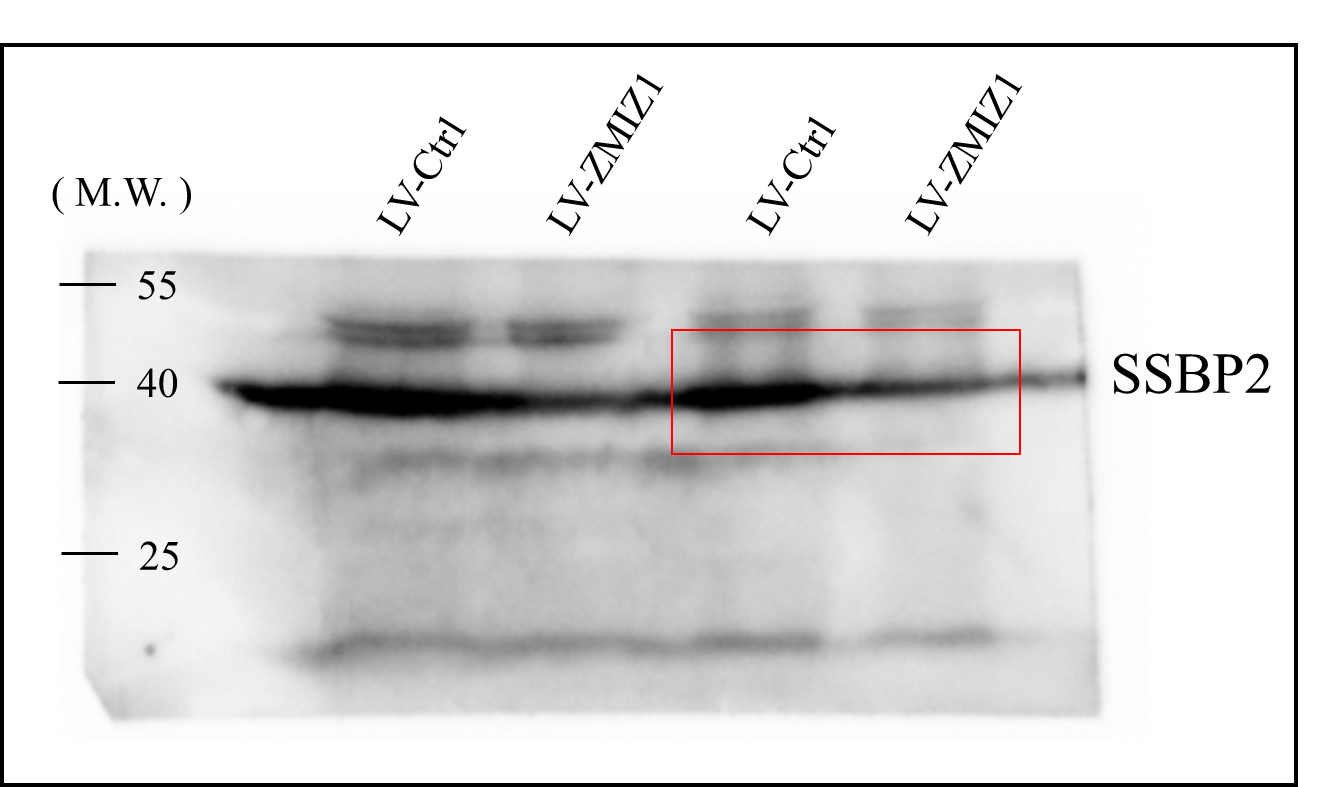
**

**Figure 3 C**

**
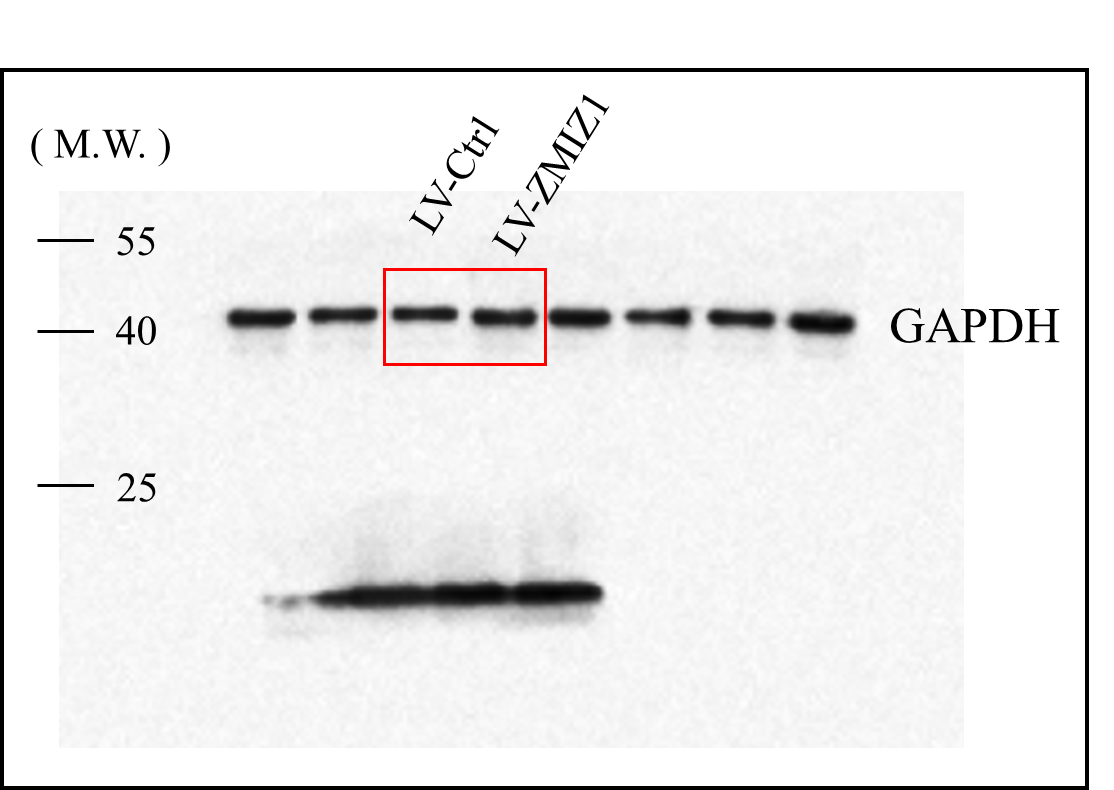
**

**
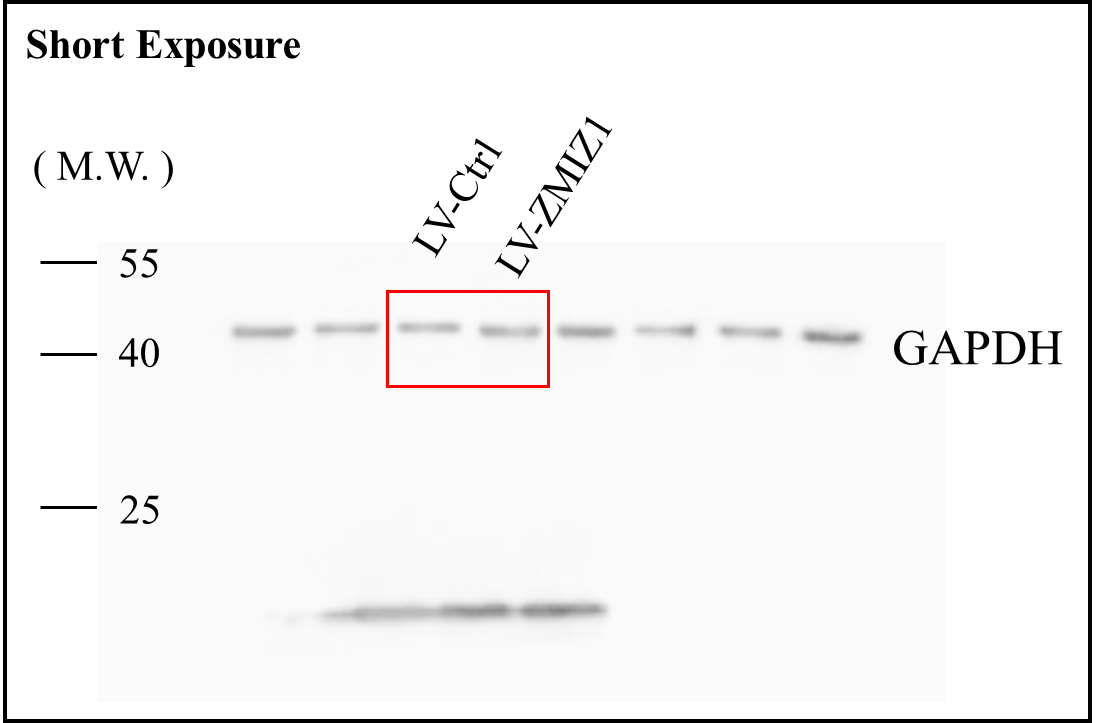
**

**Figure 4G**

**
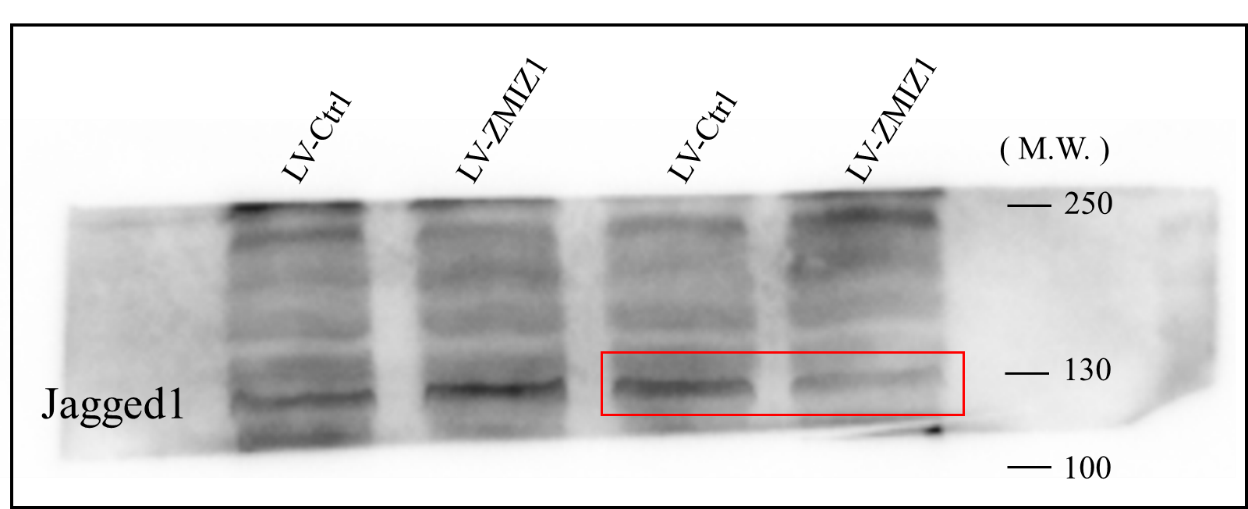
**

**
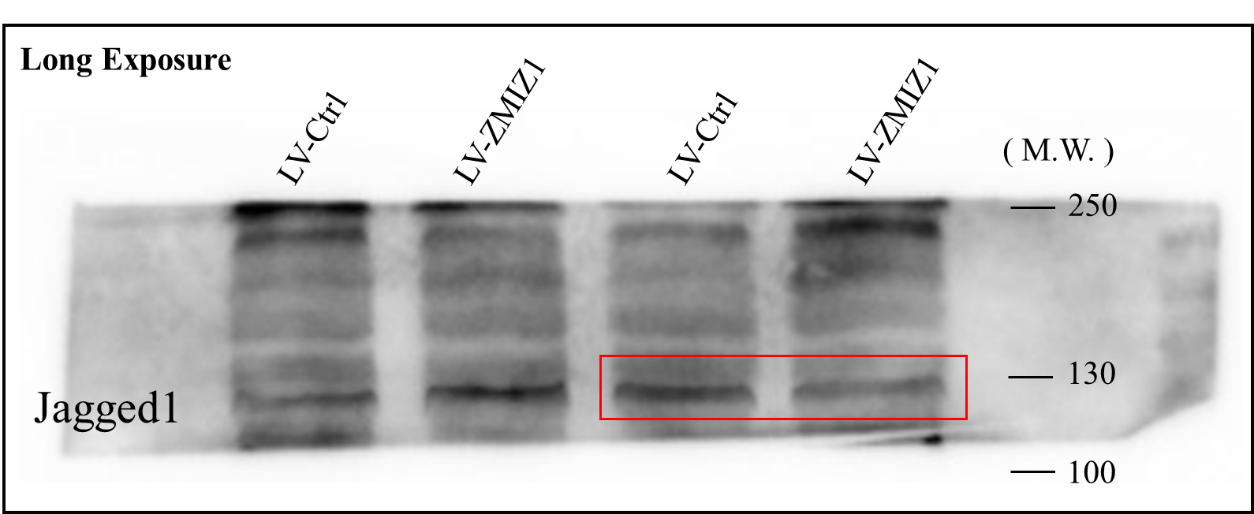
**

**Figure 4G**

**
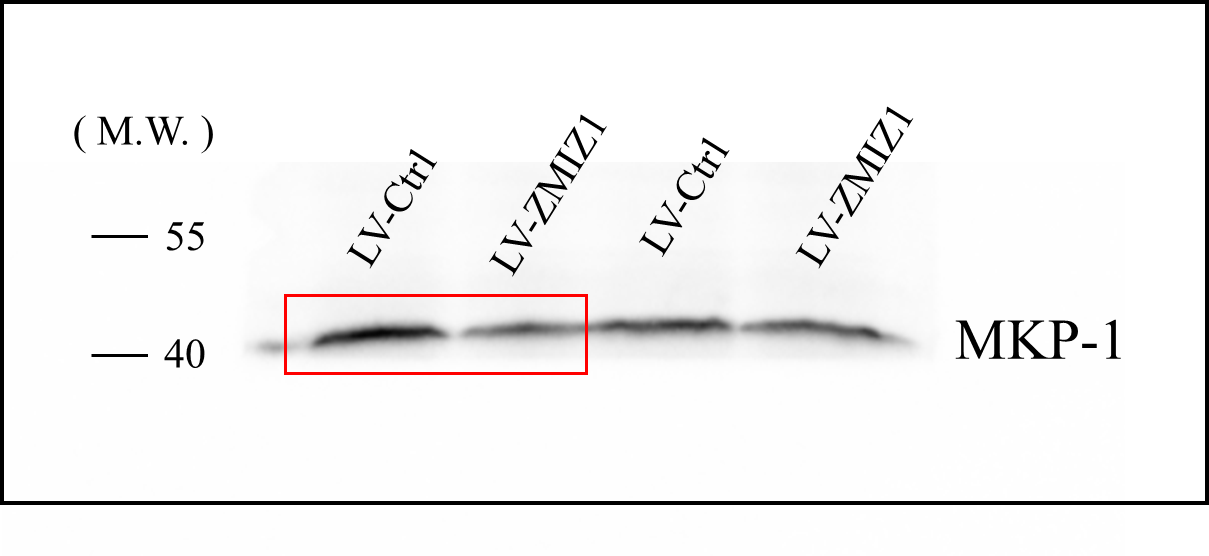

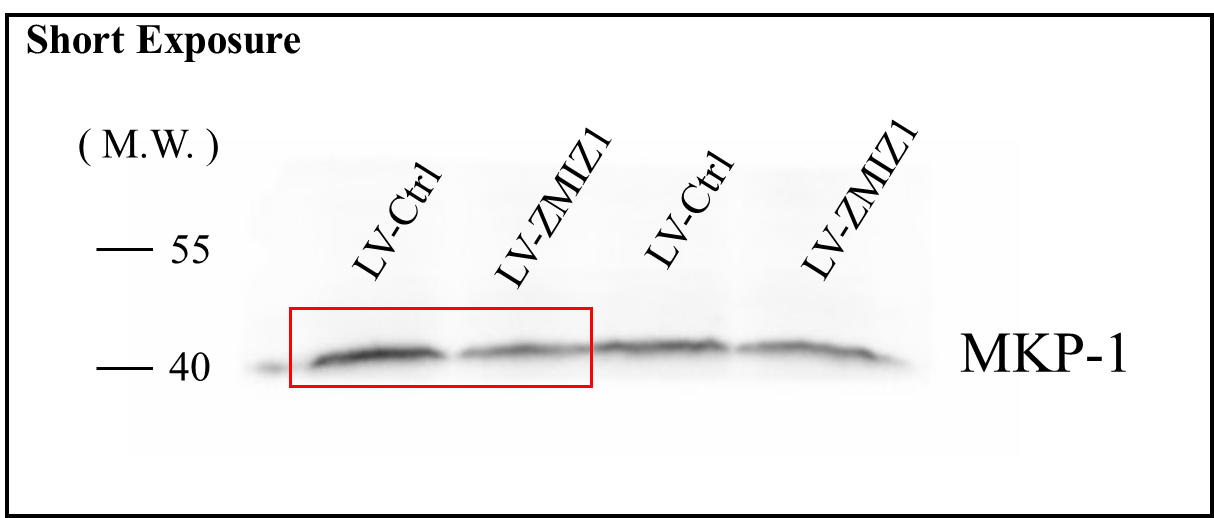
**

**
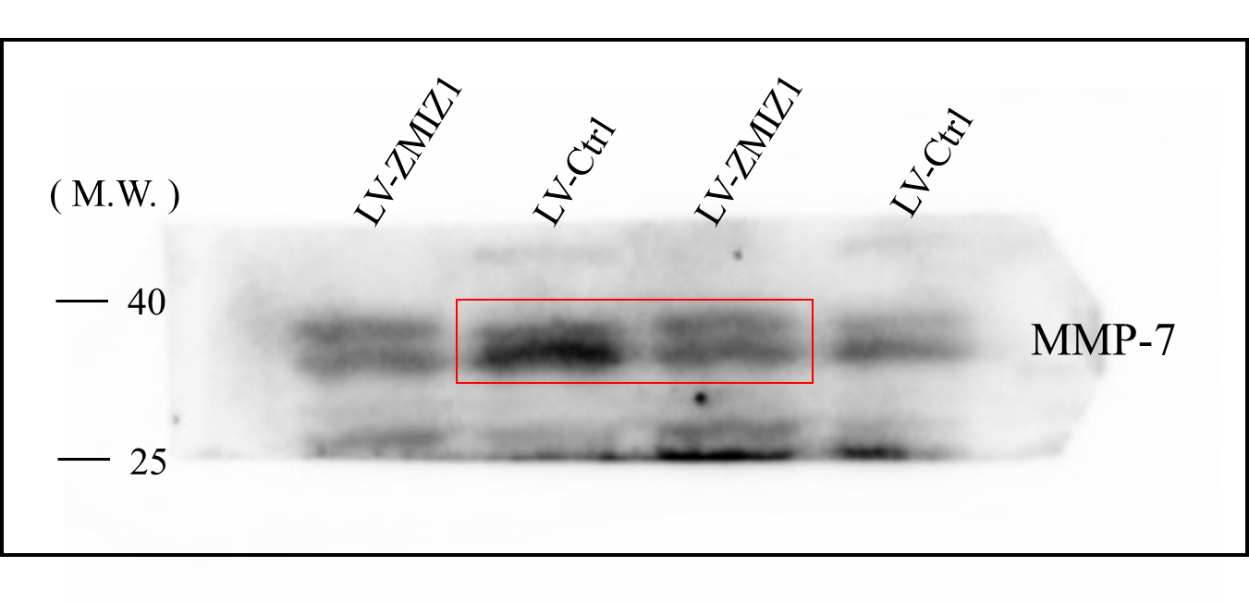

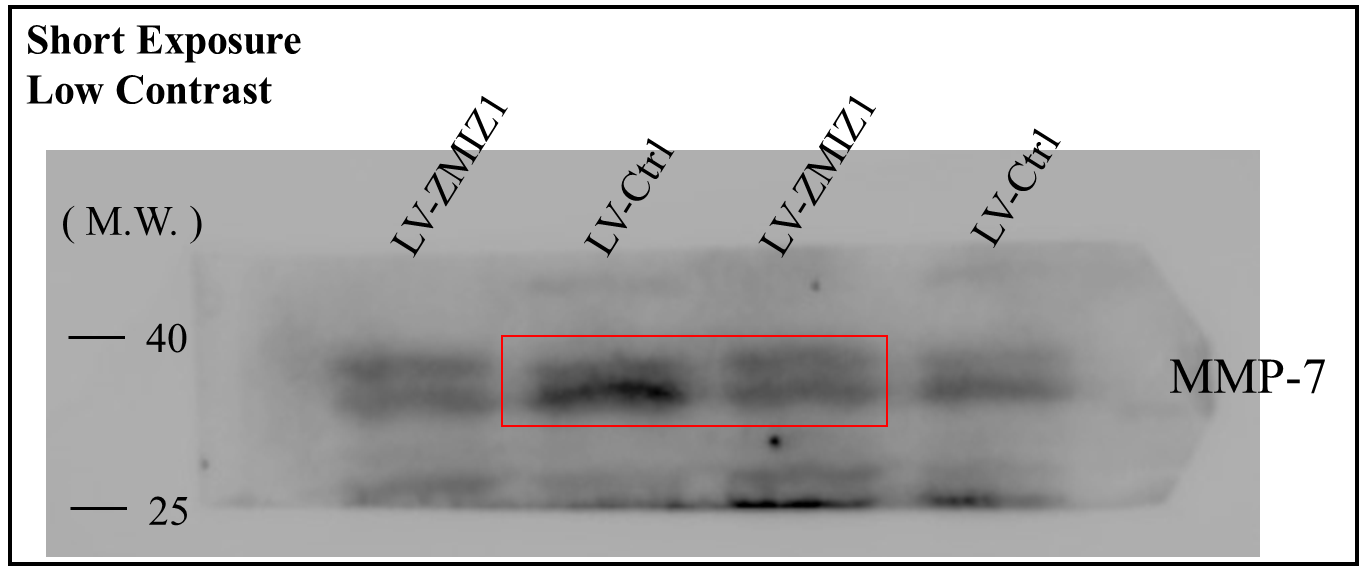
**

**Figure 4G**

**
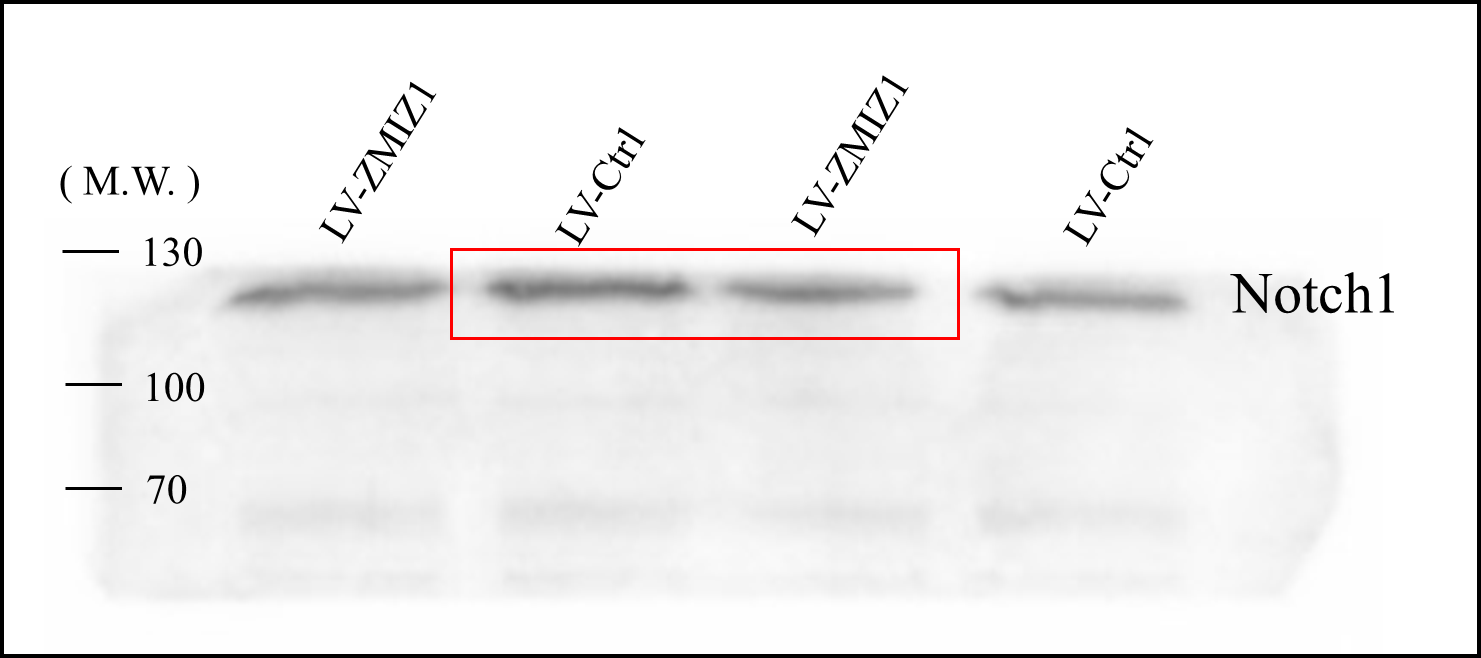
**

**
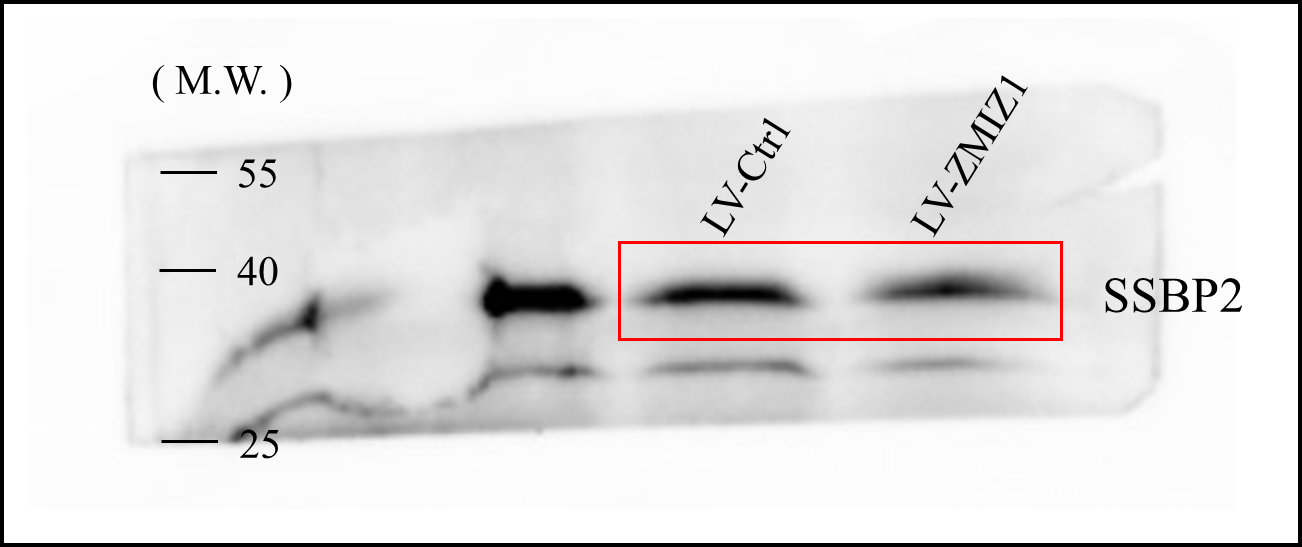
**

**Figure 4G**

**
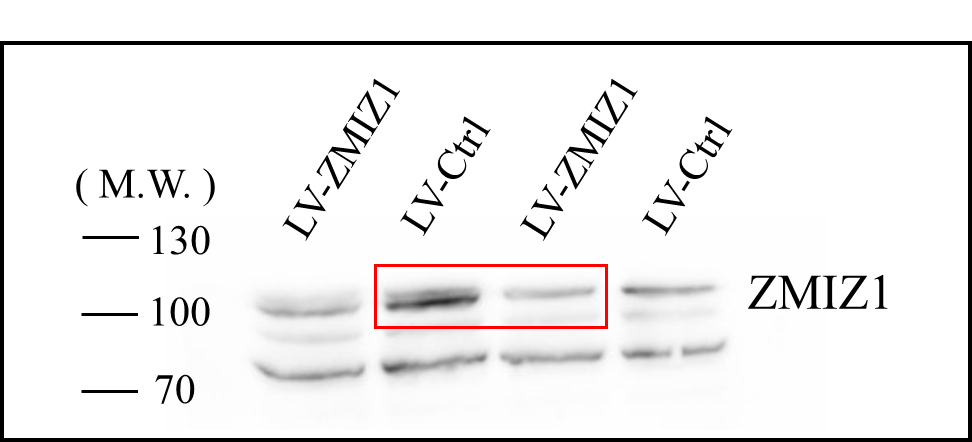

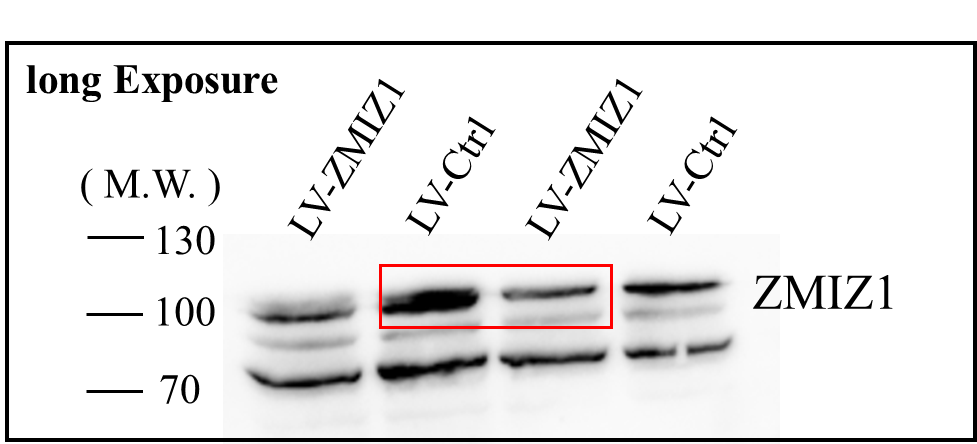
**

**Figure 4G**

**
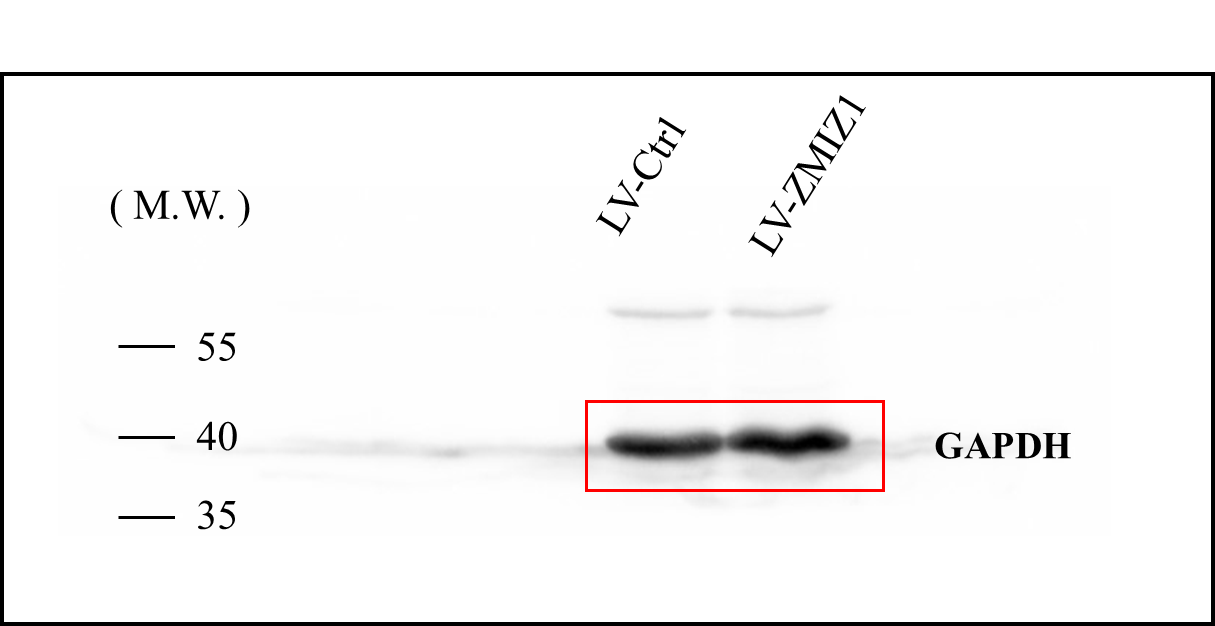

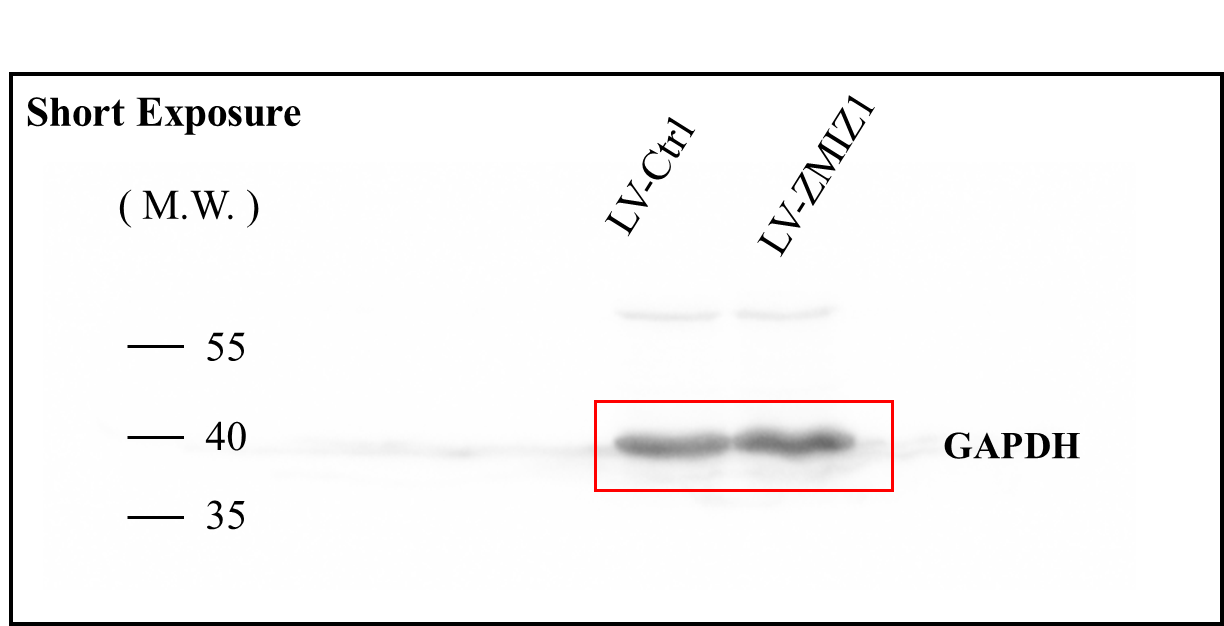
**
